# Functional development and regeneration of hair cells in the zebrafish lateral line

**DOI:** 10.1113/JP281522

**Published:** 2021-07-09

**Authors:** Katherine Hardy, Ana E. Amariutei, Francesca De Faveri, Aenea Hendry, Walter Marcotti, Federico Ceriani

**Affiliations:** 1 Department of Biomedical Science, University of Sheffield, Sheffield, S10 2TN, UK; 2 Sheffield Neuroscience Institute, University of Sheffield, Sheffield, S10 2TN, UK

## Abstract

Hair cells are mechanosensory receptors responsible for transducing auditory and vestibular information into electrical signals, which are then transmitted with remarkable precision to afferent neurons. Different from mammals, the hair cells of lower vertebrates, including those present in the neuromasts of the zebrafish lateral line, regenerate following environmental or chemical insults. Here we investigate the time-course of regeneration of hair cells *in vivo* using electrophysiology, 2-photon imaging and immunostaining applied to wild-type and genetically-encoded fluorescent indicator zebrafish lines. Functional hair cells drive spontaneous action potentials in the posterior lateral line afferent fibres, the frequency of which progressively increases over the first 10-days post-fertilization (dpf). Higher firing-rate fibres are only observed from ~6 dpf. Following copper treatment, newly formed hair cells become functional and are able to drive APs in the afferent fibres within 48 hours in both early-larval (≤8 dpf) and late-larval (12-17 dpf) zebrafish. However, the complete functional regeneration of the entire neuromast is delayed in late-larval compared to early-larval zebrafish. We propose that while individual regenerating hair cells can rapidly become active, the acquisition of fully functional neuromasts progresses faster at early-larval stages, a time when hair cells are still under development. At both ages, the afferent terminals in the regenerating neuromast appear to make initial contact with supporting cells. The ablation of the lateral line afferent neurons prevents the timely regeneration of supporting cells and hair cells. These findings indicate that the afferent system is likely to facilitate or promote the neuromast regeneration process.

## Abbreviations

dpf: day post-fertilization
hpt: hour post-treatment
PLLg: posterior lateral line ganglion
CV: coefficient of variation
MET: mechanoelectrical transducer
APs: action potentials

## Introduction

Damage of the auditory hair cells can result from a wide range of factors including environmental and chemical insults, genetic predisposition and ageing. While humans and other mammals have limited or no hair cell regenerative abilities, lower vertebrates such as the zebrafish can fully regenerate them following damage (e.g. Corwin & Obholzer, 1997; Kelley, 2003; Rubel *et al*. 2013). In the zebrafish lateral line, the hair cell regeneration process is rapid (Harris *et al*. 2003; López-Schier & Hudspeth, 2006; Hernández *et al*. 2007), with different populations of supporting cells serving as progenitors for the newly formed hair cells (Thomas & Raible, 2019). Although some of the mechanisms leading to hair cell regeneration in the lateral line have been identified (e.g. Ackinson *et al*. 2015; Monroe *et al*. 2015; Thomas *et al*.2015), little is known about the temporal profile defining the ability of hair-cells and their afferent innervation to transduce spontaneous and mechanical-induced responses during regeneration.

Zebrafish, use the lateral line to signal hydrodynamic variations and water currents around the body (Dijkgraaf, 1963; Kroese & Schellart 1992). The lateral line includes the anterior (head) and posterior (tail) regions that, by sensing low-frequency signals (of up to 200 Hz: Ghysen & Dambly-Chaudiere, 2004), mediate several behavioural tasks such as schooling, prey capture, predator avoidance and rheotaxis (e.g. Montgomery *et al*. 1997; Engelmann *et al*. 2000; Bleckmann & Zelick, 2009). The functional unit of the lateral line is the neuromast, which consists of hair cells clustered in a rosette-like structure on the surface of the fish (MetCalfe *et al*. 1985; Sheets *et al*. 2012; Olt *et al*. 2016). Each neuromast contains two sets of hair cells responding to opposite directional stimuli, a feature which is determined by the specular orientation of their mechanosensitive stereociliary bundle (Flock & Wersäll, 1962; López-Schier *et al*. 2004). Hair cells are innervated by bipolar afferent neurons that merge in cephalic ganglia (MetCalfe *et al*. 1985; Ghysen & Dambly-Chaudiere, 2004). Each afferent neuron receives information from hair cells present in one or multiple neuromasts, but of the same polarity (Obholzer *et al*. 2008; Nagiel *et al*. 2008; Faucherre *et al*. 2009). Primary neuromasts are initially deposited by a primordium along the length of the fish during the first two-days post-fertilization (dpf) (Pujol-Martí & López-Schier, 2013). Although hair cells are already functional at 3 dpf, the majority of them exhibit an immature biophysical profile (Olt *et al*. 2014). The proportion of mature-like hair cells largely increases over the following several days, and seems to mainly cluster at the centre of the neuromast (Olt *et al*. 2014). During this initial developmental period, afferent fibres are also under the influence of intrinsic developmental cues and undergo extensive reorganisation (Liao & Haehnel, 2012; Haehnel *et al*. 2012). Considering that most of the hair-cell regeneration studies in the zebrafish lateral line are performed at around 3-5 dpf, it is unclear whether the reported rapid time-course of regeneration is also a characteristic present at more functionally mature ages.

Here we performed electrophysiological, 2-photon imaging and immunostaining experiments to investigate the functional recovery of hair cells from larval zebrafish after chemical insult. We found that the functional and morphological regeneration of the hair cells in the lateral line occurred faster in early-than late-larval zebrafish. Despite the different time course of regeneration, the newly formed hair cells acquire functional characteristics within the first 24-hours post-copper treatment in both early- and late-larval zebrafish. We have also showed that the afferent terminals appear to innervate the regenerating neuromast devoid of hair cells, and instead make initial contact with the supporting cells. Zebrafish that underwent ablation of the posterior lateral line ganglion (PLLg) prevents the normal rapid regeneration of the supporting cells and hair cells. This indicates that the afferent system plays a key role in the timely response of the regeneration process.

## Materials and Methods

### Ethics Statement and zebrafish lines

All animal work was performed at the University of Sheffield (UK), licensed by the Home Office under the Animals (Scientific Procedures) Act 1986 (PPL_P0FBE066C) and approved by the University of Sheffield Ethical Review Committee (180626_Mar). The experimental work also conforms to the principles and regulations as described in the Editorial by Grundy (2015). Zebrafish (*Danio rerio*) were raised in petri dishes at ~28°C in an incubator until 5.2-days post fertilisation (dpf) when they were transferred to a light/dark incubator and fed powdered food. Zebrafish aged >10 dpf were maintained in tanks in the aquarium facility on a 14/10-hours light/dark cycle and fed artemia twice daily. At the end of each procedure, zebrafish were culled by immersion in a solution containing 0.1-0.4% of the anaesthetic tricaine methanesulfonate (MS-222; Henry Schein, Inc., Dumfries, UK) until unresponsive and then decapitated.

Experiments were performed using the following zebrafish lines: wild-type AB; *Tg(NeuroD:EGFP)* which express the green fluorescent protein EGFP in the afferent neurons to visualise the afferent terminals and posterior lateral line ganglion (Obholzer *et al*. 2008). *Tg(brn3c:GAL4);Tg(UAS:iGluSnFR)*, which express the glutamate sensor iGluSnFR (Marvin *et al*. 2013) in the hair cells to monitor vesicle release at the ribbon synapses; *Tg(Myosin6b:R-GECO)*, which express the red fluorescent Ca^2+^ indicator R-GECO in the hair cells (Maeda *et al*. 2014). *Tg(NBT:GCaMP3)* express the green fluorescent Ca^2+^ indicator GCaMP3 in the afferent neurons (Bergmann *et al*. 2018) and allowed us to measure Ca^2+^ entry at their terminals (Sebe *et al*. 2017).

**Figure 1:**
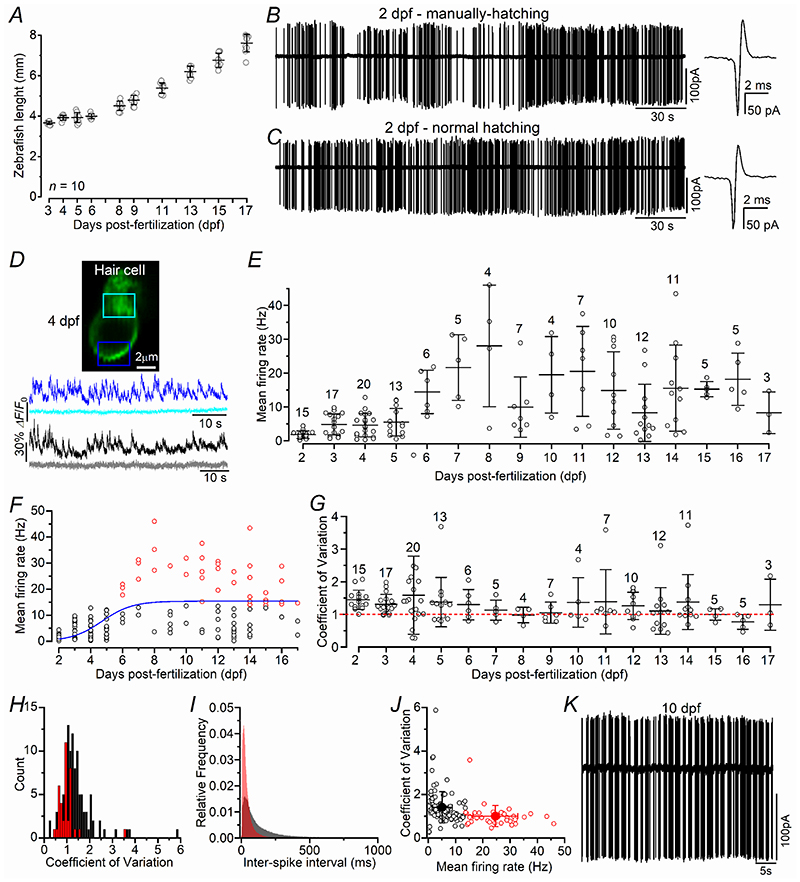
Spontaneous action potentials in the zebrafish posterior lateral line ***A***, Growth of the zebrafish (*Danio rerio*) under the husbandry conditions used at the University of Sheffield. The selected time window 2-17 dpf (10 zebrafish for each time point) reflected the age-range used for all of the following imaging and electrophysiological experiments. ***B*** and ***C***, Spontaneous action potentials (APs) from the posterior lateral line ganglion (PLLg) recorded using cell-attached voltage clamp. Recordings are from 2 days post-fertilization (dpf) zebrafish that were manually (***B***) or naturally (***C***) hatched. Manually hatched recordings were from 6 zebrafish (total of 5854 spikes over 57.2 minutes); naturally hatched: 9 zebrafish (7233 spikes over 55.0 minutes). The panels on the right show an AP on an expanded time scale. ***D***, Fluorescence image generated as an average projection of 1000 images of a lateral line hair cell from a 4 dpf *Tg(Brn3c:GAL4);Tg(UAS:iGluSnFR)* zebrafish expressing iGluSnFR (green: top panel). The bottom panel shows the iGluSnFR traces from two hair cells calculated from ROIs drawn at the synaptic pole (blue and black traces) or the neck of the cell (light-blue and grey traces). Note that the hair cells in the top image refer to the recordings depicted in the blue and light-blue traces; the image of the second hair cell (black-grey traces) is not shown. Note the high frequency signals present in the synaptic traces denoting spontaneous glutamate release (see also Supplementary Movie 1). The light traces are plotted as a visual reference of the intrinsic noise level of the recordings. ***E***, Average frequency of spontaneous APs as a function of dpf. Overall 598,004 spike from 133 PLLg (8.1 ± 2.9 min/PLLg). ***F***, The AP frequency as a function of dpf was subdivided in a low- (black circles) and a high-frequency (red circles) cluster using *k*-means clustering. The data was fitted with a Boltzman equation, with a half-peak value of 5.2 dpf. ***G***, Coefficient of variation (CV) of the spontaneous APs plotted as a function of dpf. The red dotted line indicates the CV value for a random Poisson process, which is 1. ***H***, Histogram of CV values (bin size, 0.1) for the two clusters highlighted in black and red in panel ***F***. ***I***, Histogram of individual AP inter-spike intervals of the two clusters identified in panel ***F***. ***J***, CV from each PPLg recording against their firing rate for the two groups. ***K***, Example of spontaneous APs from a PPLg of a 10 dpf zebrafish showing a bursting firing patterns (CV: 2.7; Frequency: 6.9 Hz). In this and the following Figures, data are presented as mean ± SD.

Under the husbandry conditions in place at the University of Sheffield and using information from previous studies (Kimmel *et al*. 1995; Parichy *et al*. 2009), larval zebrafish are those from ~3 dpf to ~2 weeks post-fertilization (2 wpf), juvenile zebrafish from ~2 wpf to the point at which they become sexually mature (3–6 months) (see also Olt *et al*. 2014). Considering that most of the work in this study is done within the larval stage of development (2-17 dpf), we have classified “early-larval” zebrafish as those aged between 2 and 10 dpf and “late-larval” zebrafish as those from 11 to 17 dpf. The average lengths of zebrafish maintained in our facility, and over the age range investigated, is shown in Fig. 1A.

### Patch-clamp electrophysiology

Afferent neuron recordings were obtained from the cell bodies of the posterior lateral line ganglion (PLLg). Zebrafish aged 2–5.2 dpf were briefly treated with MS-222 before being paralyzed by injecting 125 μM α-bungarotoxin (Tocris, Bristol, UK) into the heart cavity (Olt *et al*. 2014; Olt *et al*. 2016). The zebrafish (<5.2 dpf) were then transferred to a microscope chamber and immobilized onto a thin layer of sylgard using fine tungsten wire (0.015 mm diameter, Advent Research Materials, Oxford, UK). Because α-bungarotoxin injections could not be performed >5.2 dpf (zebrafish then become protected animals in the UK) older zebrafish were restrained using 2% low melting-point agarose, dissolved in E3. The agarose was also used to fix the restrained zebrafish to the bottom of the recording chamber on their lateral side. In order for the patch electrode to access the PLLg the agarose posterior to the head of the fish was gently removed. Restrained zebrafish (either using tungsten wire or agarose) were continuously superfused by a peristaltic pump with extracellular solution containing (in mM): 140 NaCl, 2.0 CaCl2, 2.0 KCl, 1.0 MgCl2, 25 NaHCO3, pH adjusted to 7.8 with NaOH. For zebrafish restrained in agarose >5.2 dpf, extracellular solution was continuously bubbled with carboxygen (95% O2 and 5% CO_2_).

The PLLg cell bodies were viewed using an upright microscope (Olympus BX51) equipped with Nomarski Differential Interference Contrast (DIC) optics (60X water immersion objective) and 15 X eyepieces. Patch pipettes were made from borosilicate capillaries (12-14 MΩ). The loose-patch configuration (35-120 MΩ) was used to record spontaneous action potential activity from the PLLg (Trapani & Nicolson, 2010; 2011; Olt *et al*. 2016). The patch pipette contained (in mM): 140 NaCl, 2.0 KCl, 2.0 CaCl2, 1.0 MgCl2, 10 Hepes-NaOH (pH 7.8). Action potentials from the PLLg were obtained at room temperature (20-24 °C) using Multiclamp 700B (Molecular Devices, USA) amplifier. Data acquisition was controlled by pClamp software using Digidata 1440A board (Molecular Devices, USA). Recordings were low-pass filtered at 2.5 kHz (8-pole Bessel), sampled at 5 kHz and stored on computer for off-line analysis (Origin: OriginLab, USA). The Mini Analysis Program (Synaptosoft Inc. NJ, USA) was used to detect spike events in loose-patch to calculate their frequency and to analyse interspike intervals (ISI). The firing rate of the action potentials was calculated as the reciprocal of the mean ISI for each cell and an indication of the spread of ISI values about the mean was obtained by calculating the coefficient of variation (CV), equal to the standard deviation (SD) divided by the mean.

### Application of the ototoxic compound copper sulphate

The ablation of hair cells and afferent terminals was achieved by the application of different concentrations of copper (II) sulphate (cat n: 451657, Sigma-Aldrich, UK), which was dissolved in plain embryonic medium E3 (in mM: 5 NaCl, 0.17 KCl, 0.33 CaCl2, 0.3 MgSO4). The different zebrafish lines (see above) at 3 dpf (early-larval) or 12 dpf (late-larval) were bath-treated with copper for 2 h at 28.5°C in the dark. Following this, the fish were washed three times in E3 and left to recover in petri dishes (<5.2 dpf) or in a non-circulating system tank (>5.2 dpf) prior to being processed for the different experiments. The use of copper was preferred to the ototoxic aminoglycoside antibiotics (e.g. neomycin and streptomycin), since under our experimental conditions streptomycin was only able to reduce the number of hair cells within a neuromast by about 70% even at very high concentrations (2 mM, Supplementary Figure 1). In addition, high concentrations of copper, but not aminoglycosides, also affected the survival of the afferent fibres and supporting cells (see Fig. 10), which allowed us to compare the time course of regeneration of different cell populations within the neuromast.

### Ablation of the afferent fibres or PLLg

For ablation of the PLL afferent nerve or the PLL ganglion, we immobilised copper-treated NeuroD fish (3 dpf) in low melting-point agarose (2%) at the bottom of a microscope chamber, which was then transferred on the stage of a dual-laser two-photon microscope (see below). The PLL was visualised using the fluorescence emission of EGFP/NeuroD under two-photon excitation (wavelength: 925 nm) using a 60X objective (Olympus). The ablation of the PLLg or the severing of the PLL afferent nerve was performed using a second un-attenuated laser beam (wavelength: 716 nm, Mai Tai BB, Spectra-Physics, USA), which was focused through the imaging objective and stirred by a pair of galvanometric mirrors across the focal plane. This second laser beam was applied in a spiral pattern on a small area (afferent severing: 425 μm^2^, 50-100 repetitions; PLLg ablation: 1500 μm^2^, 50-100 repetition) and the ablation of the PLLg or the severing of the PLL afferent nerve was visually confirmed through the EGFP fluorescence emission. Fish were then gently freed from the agarose using a pair of fine forceps and left to recover in a petri dish. Zebrafish used as the experimental control (copper-untreated) were subjected to the same experimental procedure as those treated, but omitting the copper.

### Hair bundle stimulation

The mechanical displacement of the cupula-containing the stereociliary bundles of the hair cells in the neuromast was achieved by using a fluid jet from a pipette driven by a 25 mm diameter piezoelectric disc (Erickson *et al*. 2017; Jeng *et al*. 2021; Carlton *et al*. 2021). The fluid jet pipette tip had a diameter of ~30 μm and was positioned at ~100 μm from the cupula. The distance of the pipette tip from the cupula was adjusted to elicit a maximal bundle displacement. Mechanical stimuli were applied as steps (filtered at 1 kHz, 8-pole Bessel).

### Immunofluorescence staining and light microscopy

Copper-treated and control zebrafish were anesthetized in 0.01% (early-larval fish) and 0.04% (late-larval fish) MS-222 for 10 minutes (Olt *et al*. 2016), washed in phosphate-buffered saline (PBS) and incubated in 4% paraformaldehyde for 1 hr at room temperature (RT, early-larval zebrafish) or 3 hrs at 4°C (late-larval fish). Zebrafish were then washed 3 x 10 minutes in PBS and mounted on slides using Vectashield (Vector Laboratories, cat n: H-1700). For the zebrafish requiring further immunostaining with specific antibodies, following the wash in PBS fish were washed a further 3 x 10 minutes in PBS + 0.2% Triton X-100 (Tx-100). Subsequently, zebrafish were incubated in either 5% (<5.2 dpf) or 1% (>5.2 dpf) bovine serum albumin (BSA) for 1 hr at RT. Primary and secondary antibodies were diluted in PBS 0.2% Tx-100 with either 5% or 1% BSA. Zebrafish were incubated overnight at 4°C in the following primary antibodies: anti-CtBP (1:1000, mouse IgG2, Santa Cruz, cat n: sc-55502), anti-Sox2 (1:100, rabbit, polyclonal, GeneTex, cat n: GTX124477) and anti-Myosin-VI (1:200, rabbit, polyclonal, Proteus Bioscience, cat n: 25-6791). The anti-CtBP antibody recognizes both CtBP2 and CtBP1, which localize to hair cell presynaptic ribbons in zebrafish lateral line (Sheets *et al*. 2014; Lv *et al*. 2016; Kindt & Sheets, 2018). The following day, zebrafish were washed 3 x 10 minutes in PBS, 3 x 10 minutes in PBS + 0.2% Tx-100 and then once more in PBS. Zebrafish were then incubated at RT for 1 hr (<5.2 dpf) or 2 hrs (>5.2 dpf) with the following secondary antibodies: Alexa Fluor 647 (1:1000, goat anti-mouse IgG2a, Invitrogen, cat n: A21245) and Alexa Fluor 647 (1:1000, goat anti-rabbit IgG, Invitrogen, cat n: A21245). Finally, zebrafish were washed 3 x 10 minutes in PBS and mounted on slides using Vectashield. For these experiments, zebrafish were investigated up to 17 dpf since in older fish the transgenic expression of EGFP and R-GECO was difficult to detect.

The quantification of the number of hair cells, supporting cells, CtBP puncta and afferent terminals was performed by using z-stack images captured with either a Nikon A1 confocal microscope or a Zeiss LSM 880 with AiryScan for super-resolution confocal microscopy from the Wolfson Light Microscope Facility at the University of Sheffield. Afferent terminals were identified by the presence of well-defined enlargements innervating the neuromast. Images were taken from the first 5 posterior lateral line neuromasts for zebrafish up to 5 dpf, and neuromasts 1-8 for older ages. Images and z-stacks were processed using ImageJ analysis software.

### Two-photon imaging and displacement of the hair cell stereociliary bundles

Hair cell recordings were performed from the primary neuromasts (L2–L4) originating from the first primordium (primI) (Pujol-Martí & López-Schier, 2013). Calcium and glutamate signals in hair cells and afferent terminals were recorded using a two-photon laser-scanning microscope (Bergamo II System B232, Thorlabs Inc., USA) based on a mode-locked laser system operating at 925 nm, 80 MHz pulse repetition rate, <100 fs pulse width (Mai Tai HP DeepSee, Spectra-Physics, USA). Images were captured with a 60X objective (LUMFLN60XW, Olympus, Japan) using a GaAsp PMT (Hamamatsu) coupled with a 525/40 bandbass filter (FF02-525/40-25, Semrock). Images were analysed offline using custom built software routines written in Python (Python 3.7, Python Software Foundation) and ImageJ (NIH). Ca^2+^ signals were measured as relative changes of fluorescence emission intensity (*ΔF/F*_0_). Images were acquired at 15 (512 x 512 pixels, Ca^2+^ imaging) or 396 (256 x 32 pixels, when using the *iGluSnFR* zebrafish) frames per second. Signals from individual ROI were smoothed offline using a Savitzki-Golay filter (windows size:11; polynomial order: 1). Traces were normalized to baseline fluorescence (*F*_0_) which was calculated as the 5th percentile of the fluorescence values of the entire trace.

### Statistical analysis

Statistical comparisons of means were made by Student’s two-tailed *t*-test or, for multiple comparisons, analysis of variance (one-way or two-way ANOVA followed by an appropriate post-hoc test: Tukey’s or Sidak’s post-test) was applied. *P*<0.05 was selected as the criterion for statistical significance. Mean values are quoted in text and figures as mean ± SD. Statistical analysis was performed using Prism 8.0 (GraphPad Prism Software, Inc., San Diego, CA, USA) and R (R foundation for Statistical Computing, R Core Team 2020).

## Results

### Spontaneous action potential activity in the afferent fibres

Action potential (AP) activity has been shown to occur in the lateral line afferent fibres at around 5 days post-fertilization (dpf) (e.g. Trapani & Nicolson, 2011; Liao & Haehnel, 2012; Levi *et al*. 2014), a time when the zebrafish is swimming and thus receiving sensory inputs from the surrounding environment. Therefore, we investigated whether swimming behaviour is required for the onset of spontaneous AP activity by performing experiments starting from 2 dpf, which is a time when zebrafish normally hatch from the chorion (Kimmel *et al*. 1995). Extracellular loose-patch recordings from the cell body of the neurons of the posterior lateral line ganglion (PLLg, Trapani & Nicolson, 2010; 2011; Olt *et al*. 2016) were performed from prematurely manually-hatched and swimming zebrafish. We found that PLLg neurons from manually-hatched zebrafish, with no prior swimming experience, already exhibited spontaneous AP activity with a mean frequency (1.63 ± 1.45 Hz, *n* = 6, Fig. 1B) that was not significantly different to that recorded from aged-matched swimming fish that hatched naturally (1.89 ± 0.95 Hz, *n* = 9, Fig. 1C, *P* = 0.6812, *t*-test). These data indicate that the onset of spontaneous firing activity does not require sensory inputs due to the swimming behaviour. This AP activity is likely to be driven by the spontaneous release of glutamate from hair cells (Fig. 1D; Supplementary Movie 1).

In the UK zebrafish become protected from the day of independent feeding at larval stage, which at 28.5 °C holding temperature corresponds to 5.2 dpf. Therefore, while *in vivo* experiments from zebrafish up to 5.2 dpf were done using pinned down zebrafish injected with the neuromuscular blocker α-bungarotoxin (Trapani & Nicolson, 2011; Levi *et al*. 2015; Olt *et al*. 2014), at older ages zebrafish were restrained in 2% agarose. However, we found that the different restraining method had no influence on the afferent AP firing activity, since its frequency from pinned-down 4 dpf zebrafish (4.6 ± 3.5 Hz, *n* = 20) was not significantly different to that recorded in aged-matched agarose-restrained zebrafish (6.6 ± 2.6 Hz, *n* = 6, *P* = 0.2050, *t*-test).

The neuromasts and afferent fibres of the zebrafish lateral line undergo extensive growth and reorganisation during larval stages (Liao & Haehnel, 2012; Haehnel *et al*. 2012; Olt *et al*. 2014), which may be essential for the fine tuning of the sensitivity to movement direction. Therefore, we investigated whether the characteristics of afferent spontaneous activity would also change with age. We found that the mean frequency of spontaneous APs significantly increased with age (*P* < 0.0001, one-way ANOVA, Fig. 1E). We performed unbiased classifications of afferent neurons based on their mean firing rate using k-means clustering with k=2 (Fong *et al*. 2014). Based on the mean frequency rate, PLLg neurons were subdivided into a low-frequency cluster (black circles: 5.12 ± 3.78 Hz, *n* = 106, 2-17 dpf), which was present throughtout the age-range tested, and a high-frequency cluster that only appeared from around 6 dpf (red circles: 24.63 ± 8.19 Hz, *n* = 38, *P* < 0.0001, *t*-test, Fig. 1F). We then used the coefficient of variation as a quantitative measure of regularity for spontaneous spike firing (Sonntag *et al*. 2009; Jones & Jones, 2000). The coefficient of variation (CV) for a random Poisson process is 1; values <1 indicate more regular activity and >1 indicates irregular or bursting activity. The average CV across the entire age-range was 1.30 ± 0.69 (*n* = 144) and did not change significantly over the age-range tested (*P* = 0.8735, one-way ANOVA, Fig. 1G). When the CV data was divided into the two clusters identified with the *k*-mean analysis (Fig. 1F), we found that the firing activity of the high-frequency PLLg neurons was more regular (1.00 ± 0.49 Hz, *n* = 38) compared to low-frequency neurons (1.41 ± 0.72, *n* = 106, *P* = 0.0016, *t*-test, Fig. 1H-J). Some high-frequency neurons displayed a burst-like firing pattern (Fig. 1K).

### Hair cell regeneration following chemical-induced insult

Previous work investigating hair-cell regeneration in the zebrafish lateral line has mainly been done using morphological approaches applied to transgenic fish with hair cells tagged with fluorescent reporters (e,g, GFP- or tdTomato: Pinto-Teixeira *et al*. 2013; Monroe *et al*. 2015) or fluorescent vital dyes such as DASPEI, 4-Di-2-ASP and FM1-43FX (e.g. Harris *et al*.2003; Ma *et al*. 2008; Nigel *et al*. 2008). Although these studies have provided valuable information regarding the temporal profile of hair cell regeneration, very little is currently known about their functional regeneration.

**Figure 2:**
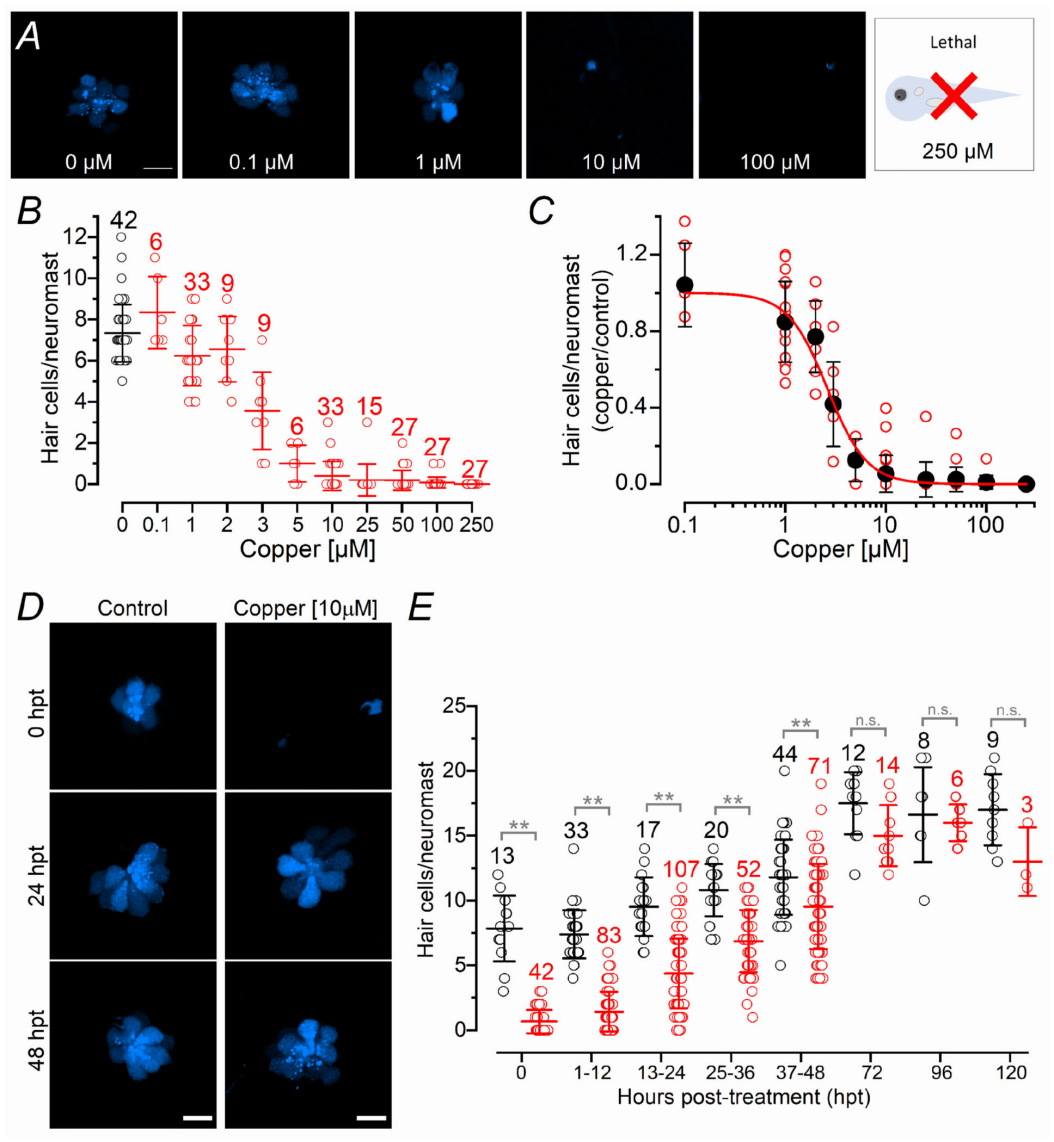
Copper ablates the posterior lateral line hair cells at μM concentrations ***A***, Confocal images of hair cells within individual neuromasts from *Tg(Myosin6b:R-GECO)* zebrafish (3 dpf) taken following a 2-hour application of varying concentrations of copper. Hair cells are labelled in blue (*Myosin6b:R-GECO*). Note that 250 μM is lethal for zebrafish. ***B***, Number of hair cells per neuromast in control zebrafish (black circles and lines) and after the application of varying copper concentrations for 2 hours (red circles and lines). ***C***, Dose–response curves for the ablation of hair cells by copper. The continuous line is the fits through the data using the Hill equation. ***D***, Confocal images of hair cells (blue) within the neuromasts of *Tg(Myosin6b:R-GECO)* zebrafish at 0, 24 and 48 hours post-treatment (hpt) with 10 μM copper for 2 hours (right columns) and aged-matched untreated control zebrafish (left columns). Scale bar: 10 μm. ***E***, Number of regenerating hair cells per neuromast as a function of hpt. Statistical significance from left to right: **P < 0.0001; n.s. *P* = 0.0702, *P* = 0.9997 and *P* = 0.1089, two-way ANOVA Sidak’s post-test. Number of neuromasts tested is shown above the data; 2-3 neuromasts per zebrafish.

We initially established the experimental protocol required to ablate the hair cells with copper, which is an ototoxic drug known to cause their death (Hernández *et al*. 2006; Mackenzie & Raible, 2012). Three-days old zebrafish were incubated for 2 hours in copper at increasing concentrations between 0.1 μM to 250 μM (Fig. 2A). The number of hair cells per neuromast, which was measured by counting the remaining labelled cells from the transgenic zebrafish *Tg(Myosin6b:R-GECO)* (see Methods), was plotted as a function of copper concentration (Fig. 2B). The concentration of copper that caused a 50% reduction in hair cell number was obtained by fitting the data with a Hill equation (IC50: 2.6 μM, Fig. 2C). Because 10 μM was the highest concentration of copper that, while ablating ~95% of hair cells, did not cause afferent fibre retraction (see Fig. 10A), it was used for most of the following regeneration experiments. Following copper-induced hair cell death, their number per neuromast gradually increased reaching a mean value comparable to that present in un-treated control zebrafish after 48 hours post-treatment (hpt) (Fig. 2D,E, *P* < 0.0001, two-way ANOVA).

### Functional regeneration of spontaneous and induced activity in the lateral line of early-larval zebrafish

**Figure 3:**
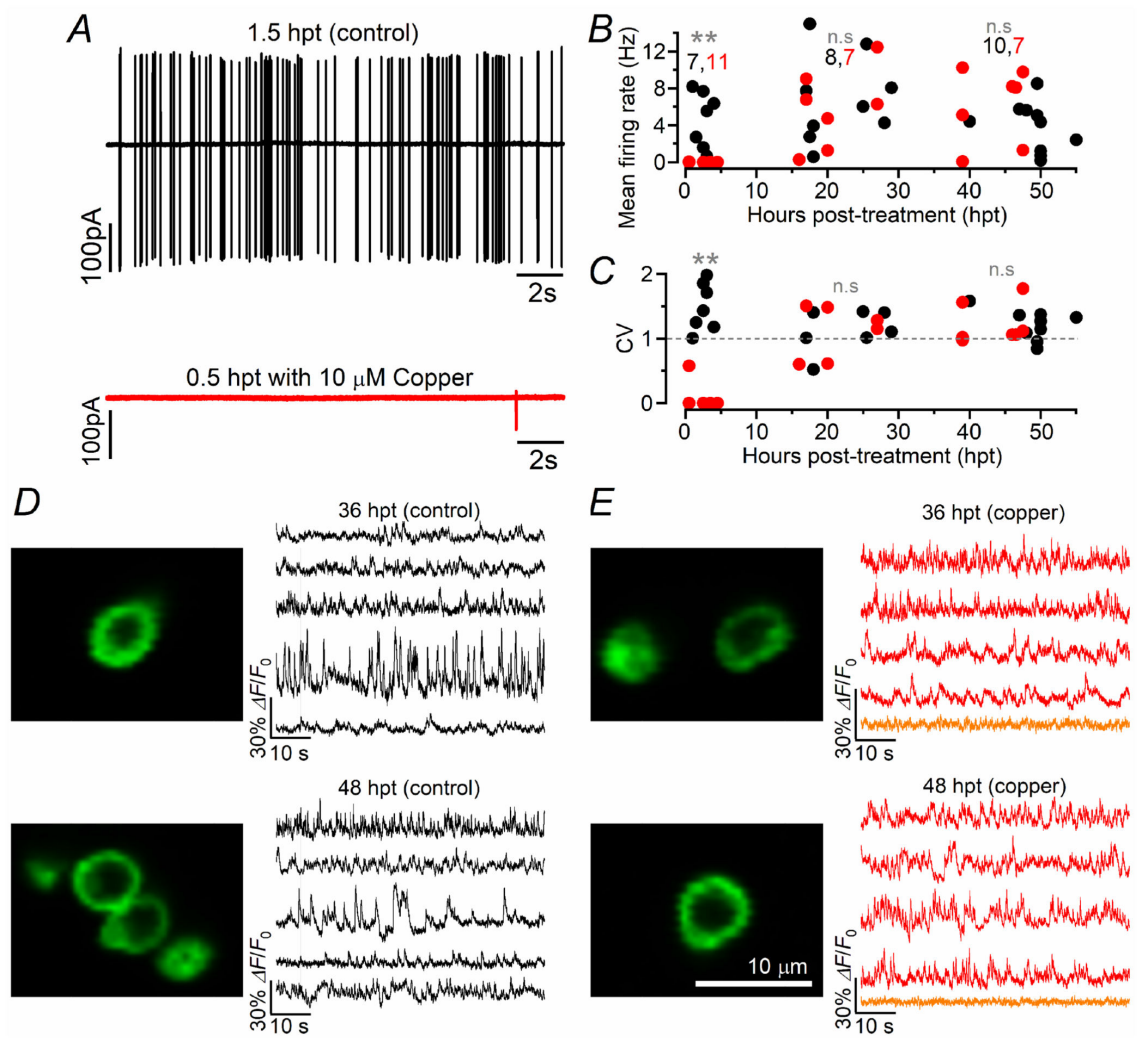
Spontaneous activity in the regenerating early-larval zebrafish posterior lateral line ***A***, Cell-attached voltage clamp recordings from a PPLg neuron of a 3 dpf zebrafish immediately after the 2-hour treatment with a solution without (top) and with (bottom) 10 μM copper. A few spontaneous APs were only present in 1 (trace shown) out of the 11 recordings from copper-treated zebrafish between 0.5 and 4.5 hpt. All zebrafish were 3 dpf at the start of the experiment (0 hpt). ***B*** and ***C***, Single data values for the frequency (***B***) and CV (***C***) of the spontaneous APs recorded from the PLLg of zebrafish undergoing hair cell regeneration after copper treatment and aged-matched control zebrafish. Statistical significance from left to right: ***B***: **P = 0.0099; n.s. *P* > 0.9999 and *P* = 0.3692; ***C***: **P = 0.0006; n.s. *P* = 0.3377 and *P* > 0.9999, two-way ANOVA Sidak’s post-test. ***D*** and ***E***, Right: fluorescence image generated as average projection of 1000 images of a lateral line hair cell from *Tg(Brn3c:GAL4);Tg(UAS:iGluSnFR)* zebrafish expressing iGluSnFR (green). Left panels show spontaneous iGluSnFR signals from the synaptic region of 3 hair cells per experimental condition. Zebrafish were investigated at 36 hpt (top panels) and 48 hpt (bottom panels). Data shown are representative images and traces from a total of 10 hair cells recorded from 4 control zebrafish (7 neuromasts) and 20 hair cells from 8 copper-treated zebrafish (18 neuromasts). Orange traces in panel ***E*** indicate hair cells lacking spontaneous iGluSnFR signals.

Functional regeneration was initially assessed by investigating the ability of newly formed hair cells to drive spontaneous AP activity in the PLLg neurons. Action potentials in the PLLg were absent in 10 out of 11 neurons from zebrafish between 0.5 and 4.5 hpt (Fig. 3A-C), but both their frequency and CV became comparable to that recorded in untreated zebrafish towards the end of the first day post-treatment (Fig. 3B and Fig. 3C, respectively). We then used the iGluSnFR reporter zebrafish line to investigate whether the regenerating hair cells were capable of neurotransmitter release. Considering that in our zebrafish line iGluSnFR is only expressed in a few hair cells within a neuromast, we found that all 10 hair cells expressing the fluorescent reporter from four untreated zebrafish showed spontaneous glutamate release (Fig. 3D), which was visible as high frequency spiking in the iGluSnFR fluorescence trace. In the copper-treated zebrafish, spontaneous glutamate release was detected in only 12 out of 20 hair cells expressing iGluSnFR (36-48 hpt, Fig. 3E), indicating the presence of hair cells at different regenerating stages at this time point.

**Figure 4:**
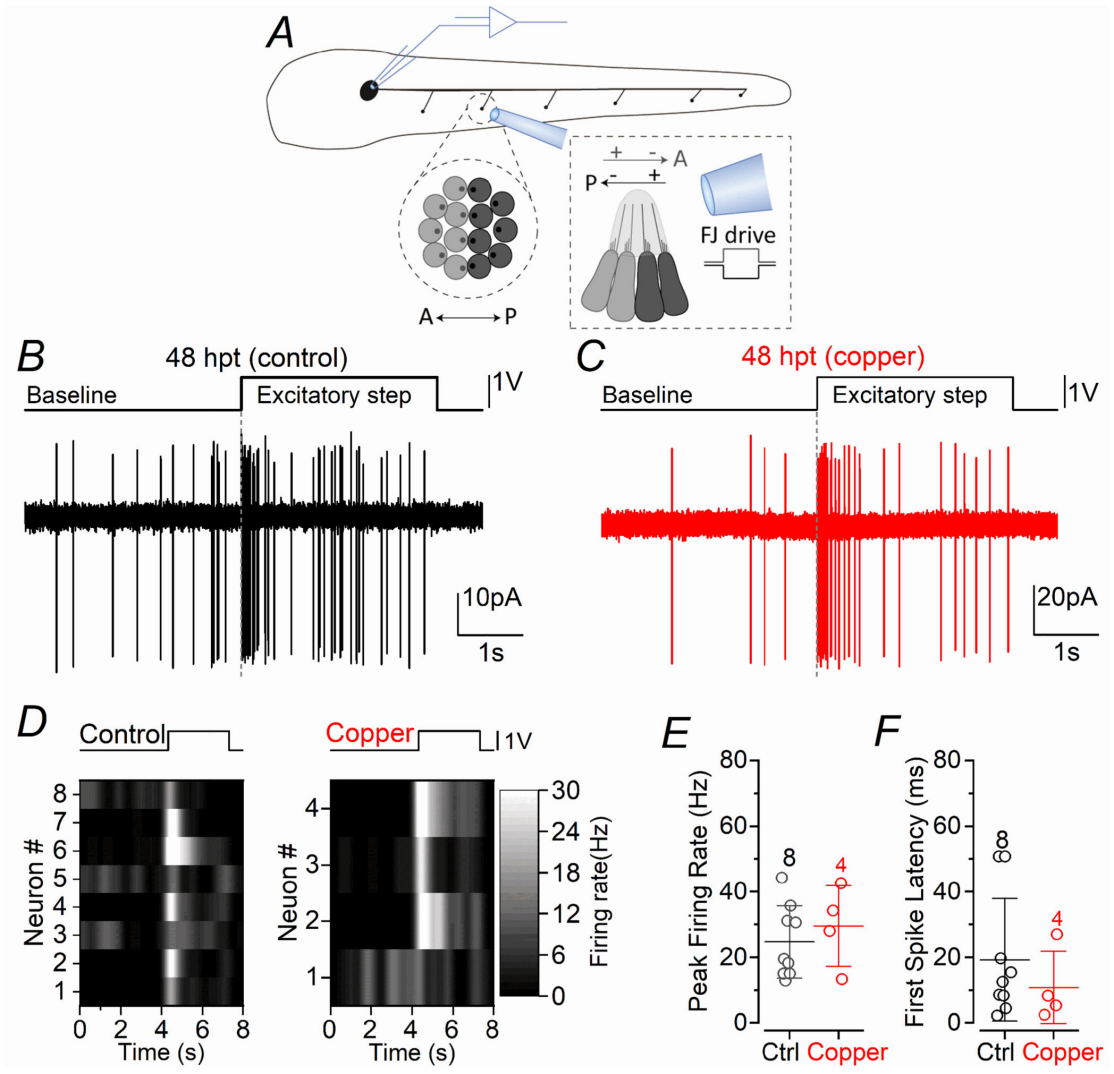
Induced firing activity in the regenerating lateral line ***A***, Schematic of the experimental set-up used to displace the mechanoelectrical transducer apparatus of the hair cells. The image shows a PLL neuromast containing hair cells of opposite polarity (light and dark grey). The hair bundles of the hair cells are displaced by saturating stimuli applied using a piezo driven fluid-jet (see Methods) while recording firing activity in the PLLg neuron connected to the neuromast. ***B*** and ***C***, Representative AP recordings from the PLLg afferent neurons while deflecting the cupula of a connected neuromast with the fluid jet (see Methods) from a control (***B***) and copper treated (***C***) zebrafish. The top trace represents the 3 s saturating driving voltage step to the piezoelectric actuator. Note the increase in firing rate at the onset of the stimulus and the subsequent adaptation. ***D***, Raster plot of individual afferent neuron activity during the application of the stimuli in the excitatory direction (top panels: driving voltage). ***E*** and ***F***, The peak firing rate (***E***) and the latency between the stimulus onset and the generation of the first spike (***F***) were not significantly different between control and copper-treated zebrafish (*P* = 0.4956 and *P* = 0.5243, *t*-test, respectively).

The ability of regenerating hair cells to respond faithfully to sensory-induced stimuli was investigated by deflecting the neuromast cupula containing the stereociliary bundles towards the excitatory direction with a fluid-jet (Fig. 4A), a procedure that gate-opens the mechanoelectrical transducer channels located at the tip of the transducing stereocilia. Excitatory cupula displacements caused the frequency of the spiking activity of the PLLg neurons of 4-5 dpf zebrafish to increase rapidly at the stimulus onset from the baseline (Fig. 4B,D). A similar increase in firing rate was also observed in aged-matched zebrafish that underwent regeneration for 48 hpt (Fig. 4C,D). The peak firing rate (Fig. 4E) and first spike latency (Fig. 4F) of the AP activity were not significantly different in the two experimental conditions.

**Figure 5:**
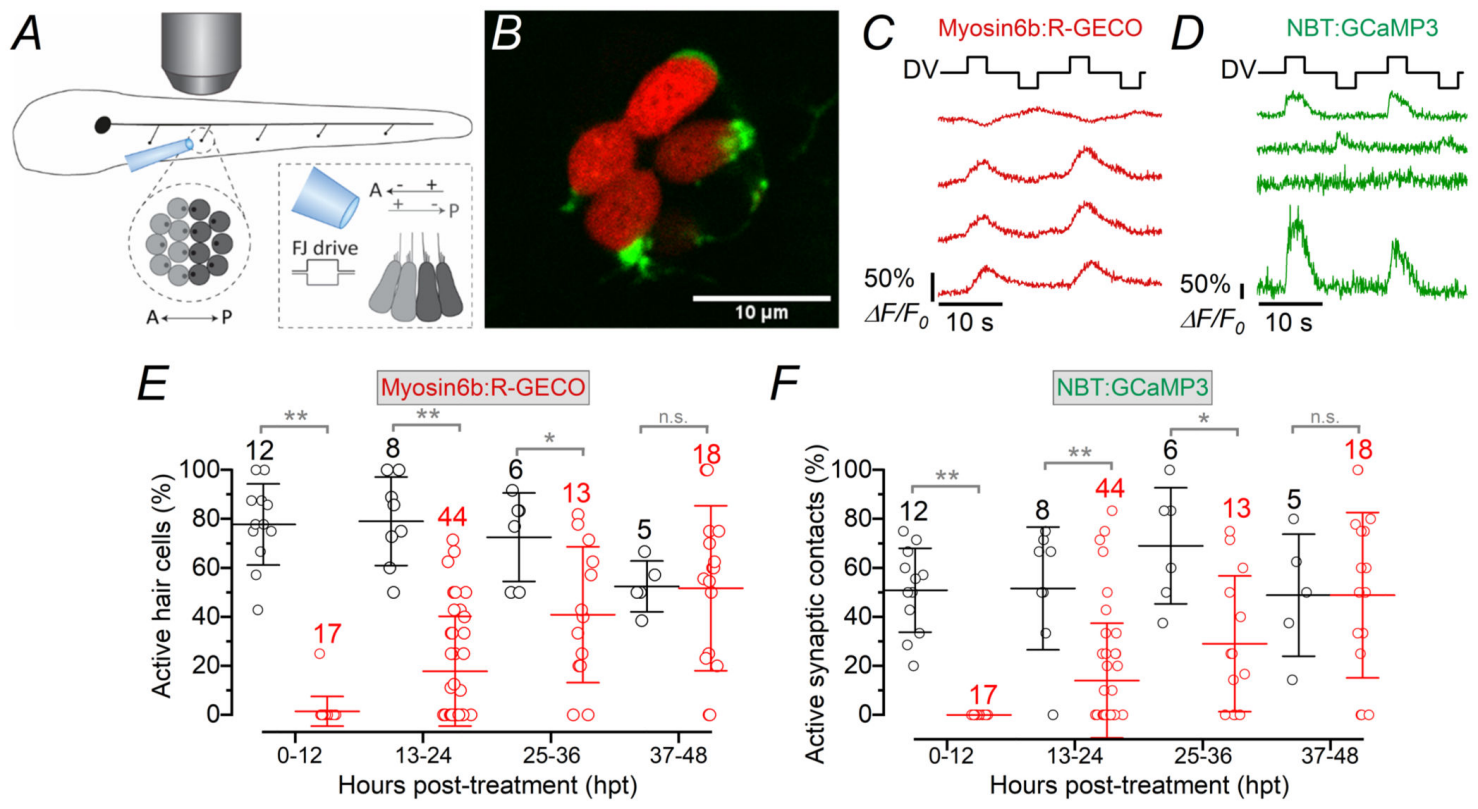
Synaptic activity in regenerating early-larval hair cells ***A***, Schematic of the experimental set-up used to displace the mechanoelectrical transducer apparatus of the hair cells. The image shows a PLL neuromast containing hair cells of opposite polarity (light and dark grey) viewed by the microscope objective (above the fish). The hair bundles of the hair cells are displaced by saturating stimuli applied using a piezo-driven fluid-jet (see Methods). Hair cells are excited by stimuli which displace their hair bundle towards the kinocilium, opening the MET channels and depolarising the membrane potential. The increase in intracellular Ca^2+^ ensues from the opening of voltage-gated Ca^2+^ channels in the plasma membrane. ***B***, Image of a 3 dpf zebrafish (*Tg(Myosin6b:R-GECO);Tg(NBT:GCaMP3)*) neuromast, in which hair cells are label red and the afferent fibres/terminals in green (see also Supplementary Movie 2). ***C***, *R-GECO* responses of four hair cells to two saturating bundle displacement stimuli showed by the driver voltage above the recordings. ***D***, Calcium responses (GCaMP3) in the afferent terminals during the same stimulation protocol that elicited hair cell responses (***C***). Note that the traces in ***C*** and ***D*** are from the same recording, but not obtained simultaneously, due to the difficulty of having several hair cells and their afferent terminals in the same focal-plane. ***E***, Percentage of hair cells showing presynaptic Ca^2+^ response during hair bundle stimulation in both control (black) and copper-treated (red) zebrafish as a function of hpt. ***F***, Percentage of afferent synaptic terminals showing GCaMP3 responses during bundle stimulation in control (black) and copper-treated (red) zebrafish as a function of hpt. Number of neuromasts tested is shown above the data; 2-3 neuromasts per zebrafish. Statistical significance from left to right: ***E***: **P < 0.0001; *P* < 0.0001; *P = 0.0204; n.s. *P* > 0.9999; ***F***: **P < 0.0001; **P = 0.0003; *P = 0.0036; n.s. *P* > 0.9999, Sidak’s post-test two-way ANOVA.

The displacement of the cupula in untreated early-larval zebrafish with a fluid-jet elicited Ca^2+^ responses in hair cells (red: Fig. 5B,C) and afferent post-synaptic activity (green: Fig. 5B,D; see also Supplementary Movie 2). After treating 3 dpf *Tg(Myosin6b:R-GECO);Tg(NBT:GCaMP3)* zebrafish with 10 μM copper, we found that the percentage of regenerating hair cells per neuromast responding with Ca^2+^ signals during the deflection of the cupula was significantly lower than that from age-matched control zebrafish (Fig. 5E, *P* < 0.0001, two-way ANOVA). The same was also found for the number of post-synaptic terminals displaying Ca^2+^ elevations during fluid-jet stimulation (Fig. 5F, *P* = 0.0100, two-way ANOVA). However, by 48 hpt, the fractions of responding hair cells and synaptic contacts were no longer significantly different from controls (*P* > 0.9999, Sidak’s post-test, for both Fig. 5E,F). Interestingly, we found that only ~50% of the post-synaptic afferent terminals within a neuromast showed Ca^2+^ signal changes during excitatory displacement of the cupula (Fig. 5F). This indicates that a large number of hair cells did not release any glutamate despite the fact that the large majority of them responded to the displacement of their hair bundles (Fig. 5E). This finding is comparable to that obtained when hair cell synaptic activity was measured in zebrafish expressing SypHy (~30%: Zhang *et al*. 2018), which is an indicator of vesicle fusion (Odermatt *et al*. 2012).

**Figure 6:**
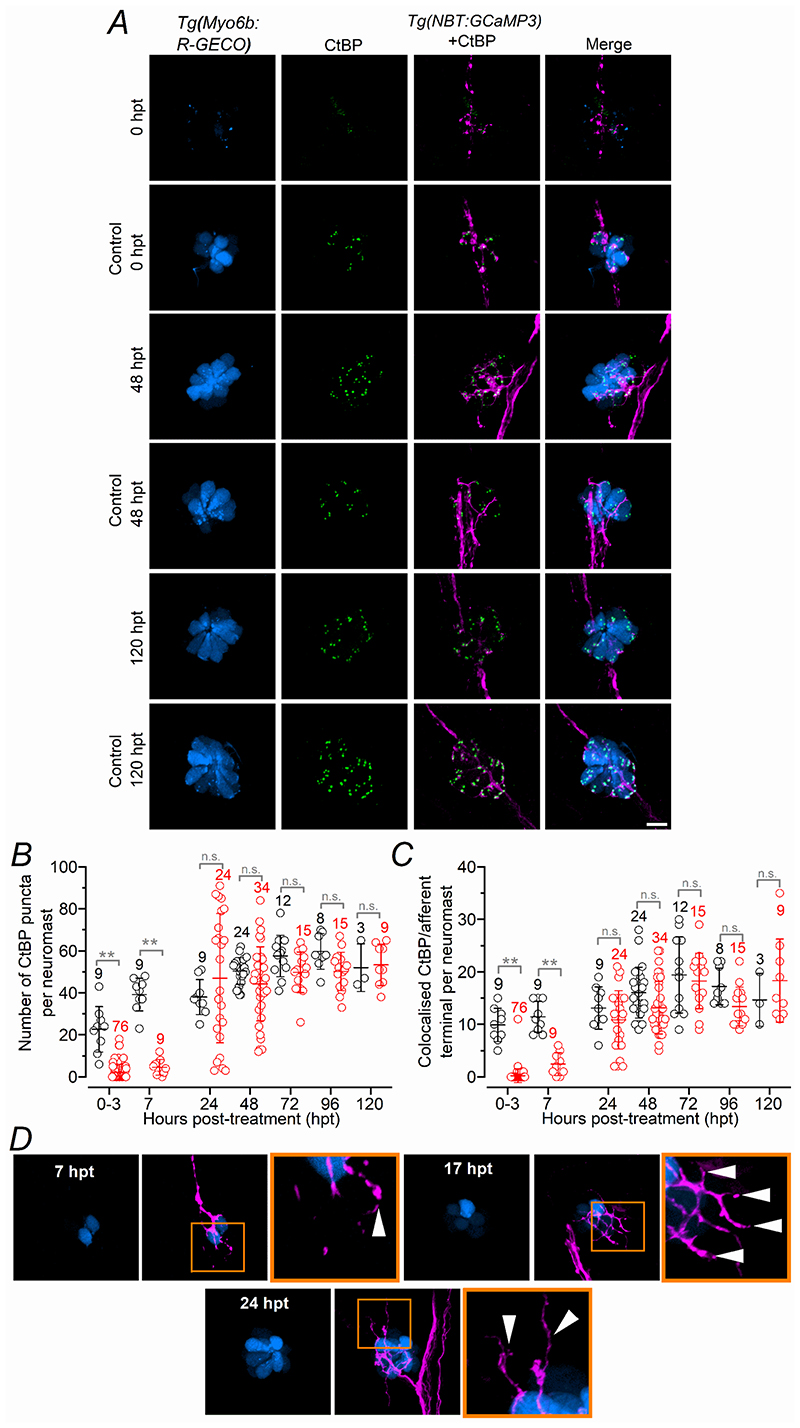
Ribbon synapses and afferent fibres in regenerating early-larval hair cells ***A***, Confocal images of hair cells (blue) and afferent fibres (magenta) within the neuromasts of *Tg(Myosin6b:R-GECO); Tg(NBT:GCaMP3)* zebrafish at 0, 48, and 120 hpt in control and copper-treated zebrafish. Experiments were done using 3 dpf zebrafish. Ribbon synapses were visualized with an antibody against the presynaptic ribbon protein RIBEYE (CtBP: green). Scale bar: 10 μm. For larger images see Supplementary Figure 4. Note that the punctate-like labelling (column: *Tg(Myosin6b:R-GECO*) in hair cells, which co-localizes with the CtBP labelling (columns: CtBP and Merge), is a characteristic of the *Tg(Myosin6b:R-GECO)* zebrafish since it was also present in the absence of the anti-CtBP antibody (see Fig. 2). ***B***, Number of CtBP puncta present in hair cells from untreated control zebrafish (black) and regenerating hair cells following copper-treatment (red) as a function of hpt. Statistical significance from left to right: **P < 0.0001; **P < 0.0001; n.s. *P* = 0.4370, *P* = 0.4465, *P* = 0.6064, *P* = 0.5057, *P* > 0.9999: two-way ANOVA Sidak’s post-test. ***C***, Number of CtBP puncta colocalized with the afferent terminals as a function of hpt. **P < 0.0001; n.s. *P* < 0.0001, *P* = 0.7407, *P* = 0.0764, *P* = 0.9889, *P* = 0.2051, *P* = 0.7763: Sidak’s post-test. Number of neuromasts tested in panel ***B*** and ***C*** is shown above the data; 2-3 neuromasts per zebrafish. ***D***, Confocal images as shown in panel ***A***, indicating the presence of afferent terminals in the absence of hair cells. Scale bar: 10 μm.

The functional recovery of synaptic function following hair cell ablation (Figs. 4, 5) was then correlated with morphological regeneration. *Tg(Myosin6b:R-GECO);Tg(NBT:GCaMP3)* zebrafish (3 dpf) were treated with 10 μM copper and then immunostained using antibodies to label the ribbon synapses (CtBP1 and CtBP2: see Methods) in hair cells of the lateral line (Sheets *et al*. 2014; Lv *et al*. 2016; Kindt & Sheets, 2018). A few CtBP puncta were evident over the few hours following copper treatment, but by 24 hpt their number was not significantly different compared to that found in untreated hair cells (Fig. 6A-C). However, during the 24-48 hpt period, the number of CtBP puncta in regenerating hair cells became very variable (Fig. 6B). A larger number of ribbons during the initial stages of hair cell regeneration has also been reported in 5 dpf zebrafish treated with the aminoglycoside neomycin (Suli *et al*. 2016), indicating the presence of some degree of synaptic refinement during regeneration. The number of CtBP puncta that colocalized with the afferent terminals was also comparable between the two experimental conditions by 24 hpt (Fig. 6C). We also observed the presence of several afferent terminals within the regenerating neuromasts that were still devoid of hair cells (Fig. 6D). The absence of visible hair cells was not due to the inability of the *Tg(Myosin6b:R-GECO)* reporter line to identify newly formed hair cells since *Myosin6b* has been shown to be expressed in hair cell precursors (Seiler *et al*. 2004), and R-GECO labels nascent hair cells well before they can be detected by antibody staining (Supplementary Figure 2).

The above data show that functional regeneration of hair cells in early-larval zebrafish can occur within 1 day from copper-induced cell death. However, due to the continuing addition of hair cells and the extensive synaptic refinements in the regenerating lateral line, it requires approximately 2 days for a neuromast to re-acquire normal functionality.

### Functional regeneration of hair cells in late-larval zebrafish requires a longer timescale than early-larval zebrafish

**Figure 7:**
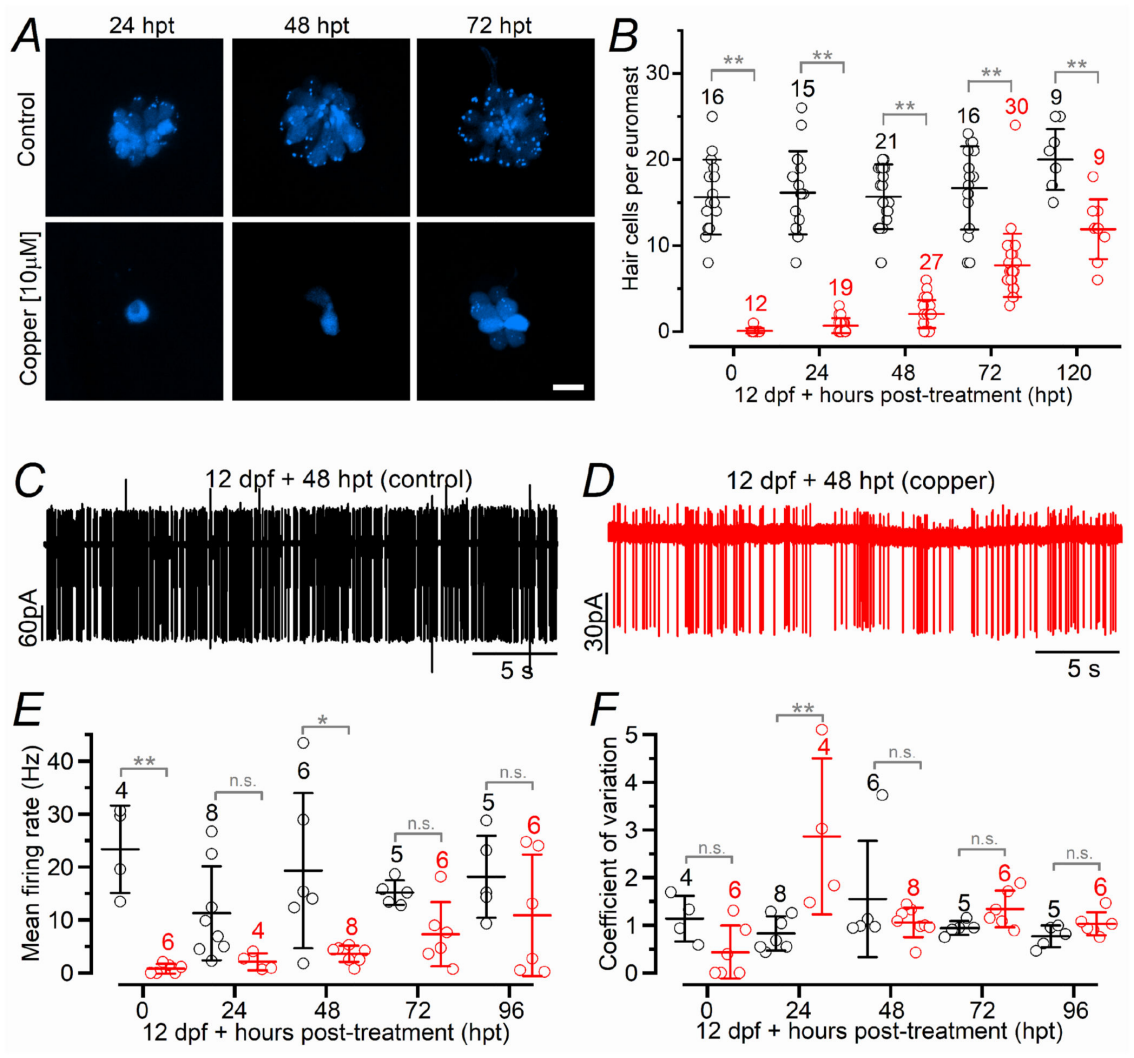
Hair cell regeneration and afferent activity in late-larval zebrafish ***A***, Confocal images of hair cells (blue) within the neuromasts of *Tg(Myosin6b:R-GECO)* zebrafish at 24, 48 and 72 hpt from copper-treated (bottom row) and control (top row) zebrafish. Experiments were done using 12 dpf zebrafish. Scale bar: 10 μm. ***B***, Number of regenerating hair cells per neuromast as a function of hpt. Statistical significance from left to right: **P < 0.0001 for all comparisons: two-way ANOVA Sidak’s post-test. ***C*** and ***D***, Spontaneous APs from the PLLg neurons. Age of the zebrafish is reported above the traces. ***E***, Frequency of spontaneous APs as a function of hpt. Significance from left to right: **P = 0.0003; n.s. *P* = 0.2768; *P = 0.0027; n.s. *P* = 0.4255; n.s. *P* = 0.5101, two-way ANOVA Sidak’s post-test. ***F***, Coefficient of variation (CV) of the spontaneous APs plotted as a function of hpt. Statistical significance from left to right: n.s. *P* = 0.4188; **P < 0.0001; n.s. *P* = 0.6057; n.s. *P* = 0.8508; n.s. *P* = 0.9727, two-way ANOVA Sidak’s post-test. In panels ***B***, ***E*** and ***F***, the number of neuromasts tested is shown above the data; 2-3 neuromasts per zebrafish.

**Figure 8:**
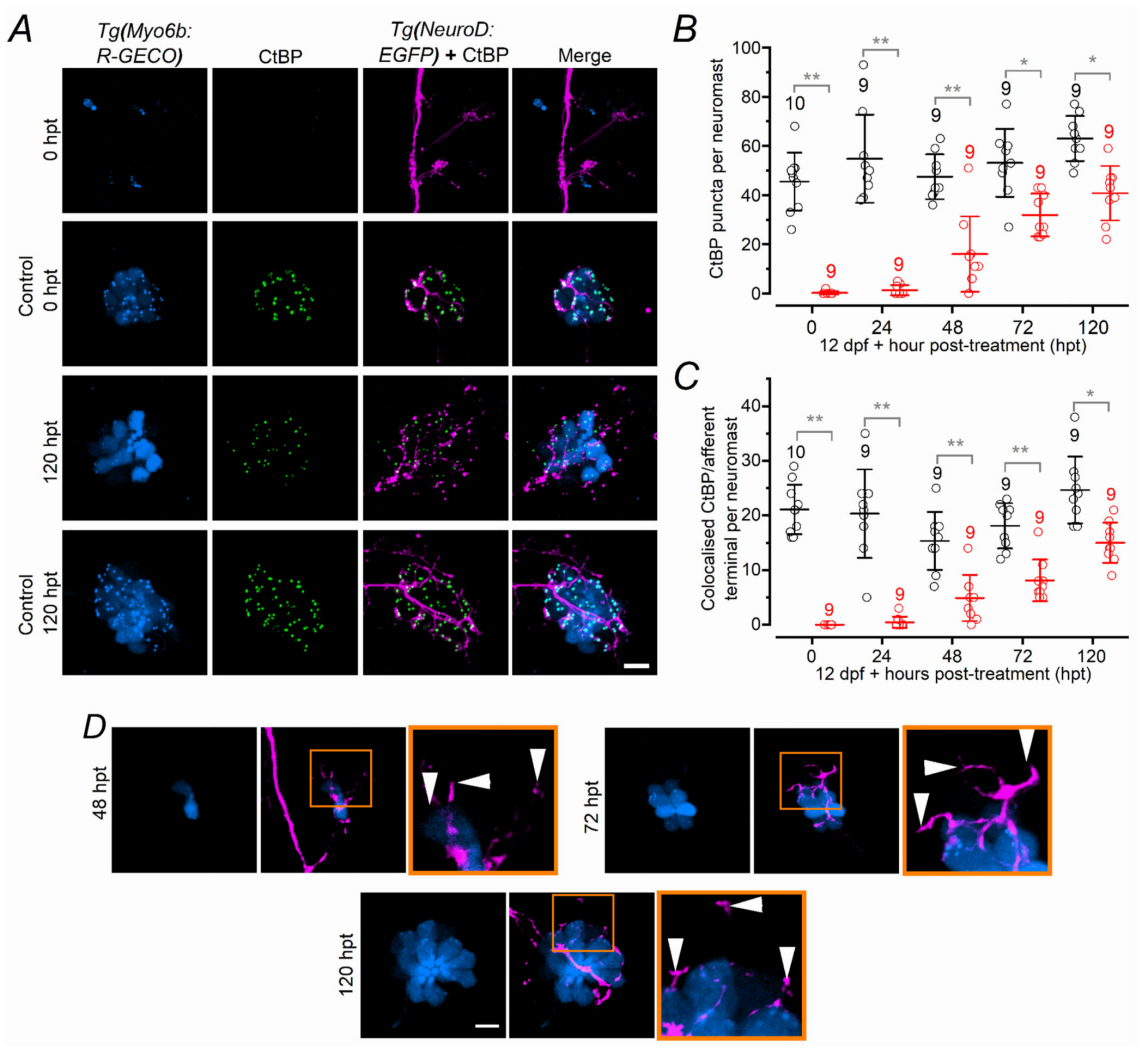
Ribbon synapses and afferent fibres in regenerating late-larval hair cells ***A***, Confocal images showing the hair cells within a neuromast from *Tg(Myosin6b:R-GECO);Tg(NeuroD:EGFP)* zebrafish obtained at 0 and 120 hpt in control and copper-treated zebrafish. Experiments were done using 12 dpf zebrafish. Ribbon synapses were visualized with the anti-CtBP antibody (green). Scale bar: 10 μm. ***B***, Number of CtBP puncta present in hair cells from the two different experimental conditions as a function of hpt. Significance from left to right: **P < 0.0001 for 0, 24 and 48 hpt; *P = 0.0007; *P = 0.0003, two-way ANOVA Sidak’s post-test. ***C***, Number of CtBP puncta colocalized with the afferent terminals as a function of hpt. **P < 0.0001 for 0, 24, 48 and 72 hpt, *P = 0.0002: two-way ANOVA Sidak’s post-test. ***D***, Confocal images as described in panel ***A***, indicating the presence of afferent terminals in the absence of hair cells. Scale bar: 10 μm.

We sought to determine whether the timescale of regeneration in the early-larval lateral line was mirrored in the late-larval zebrafish, which is when neuromasts contain a higher proportion of mature hair cells (Olt *et al*. 2014). Because of the additional ethical implications and experimental complexities when working with late-larval zebrafish, only a subset of the experiments performed in the larvae were carried out in older zebrafish. As for larvae, the treatment of late-larval zebrafish (12 dpf) with 10 μM copper for 2 hrs caused the loss of hair cells in the lateral line neuromasts. Although the number of regenerating hair cells per neuromast gradually increased between 24 and 120 hpt, it was significantly reduced compared to that present in control neuromasts over the same time period (Fig. 7A,B, *P* < 0.0001, two-way ANOVA). This finding indicates that neuromasts from late-larval zebrafish require more than 3 additional days to fully regenerate all the hair cells compared to larvae (Fig. 2E). This discrepancy is not due to the different number of hair cells present in a fully functional neuromast after copper treatment, since it was comparable between early-larval (72-120 hpt or 6-8 dpf: 15.0 ± 2.3, *n* = 23, Fig. 2E) and late-larval zebrafish (0-120 hpt or 12-16 dpf: 16.1 ± 4.4, *n* = 71, *P* = 0.2547, Fig. 7B). While at 0 hpt firing activity was absent or sporadic in PLLg neurons, at 24 hpt a more sustained activity was already evident (Fig. 7C-F), although significantly reduced in frequency compared to control zebrafish (Fig. 7E, *P* < 0.0001, two-way ANOVA). The large variability in the firing activity characteristics of control PLLg neurons (see also Fig. 1E), which is primarily due to the acquisition of high-frequency firing neurons in older zebrafish (Fig. 1F), was largely absent at least during the first few days post-copper treatment in late-larval zebrafish. A similar trend of recovery to that observed in the number of hair cells (Fig. 7B), was also present for the number of CtBP puncta per neuromast and those that colocalized with the afferent terminals (Fig. 8A-C), which were significantly reduced compared to those found in control zebrafish over the entire range tested (*P* < 0.0001, two-way ANOVA, for both comparisons). As shown for the early-larval zebrafish (Fig. 6D), we observed the formation of several afferent projections within the regenerating neuromasts without hair cells (Fig. 8D).

**Figure 9:**
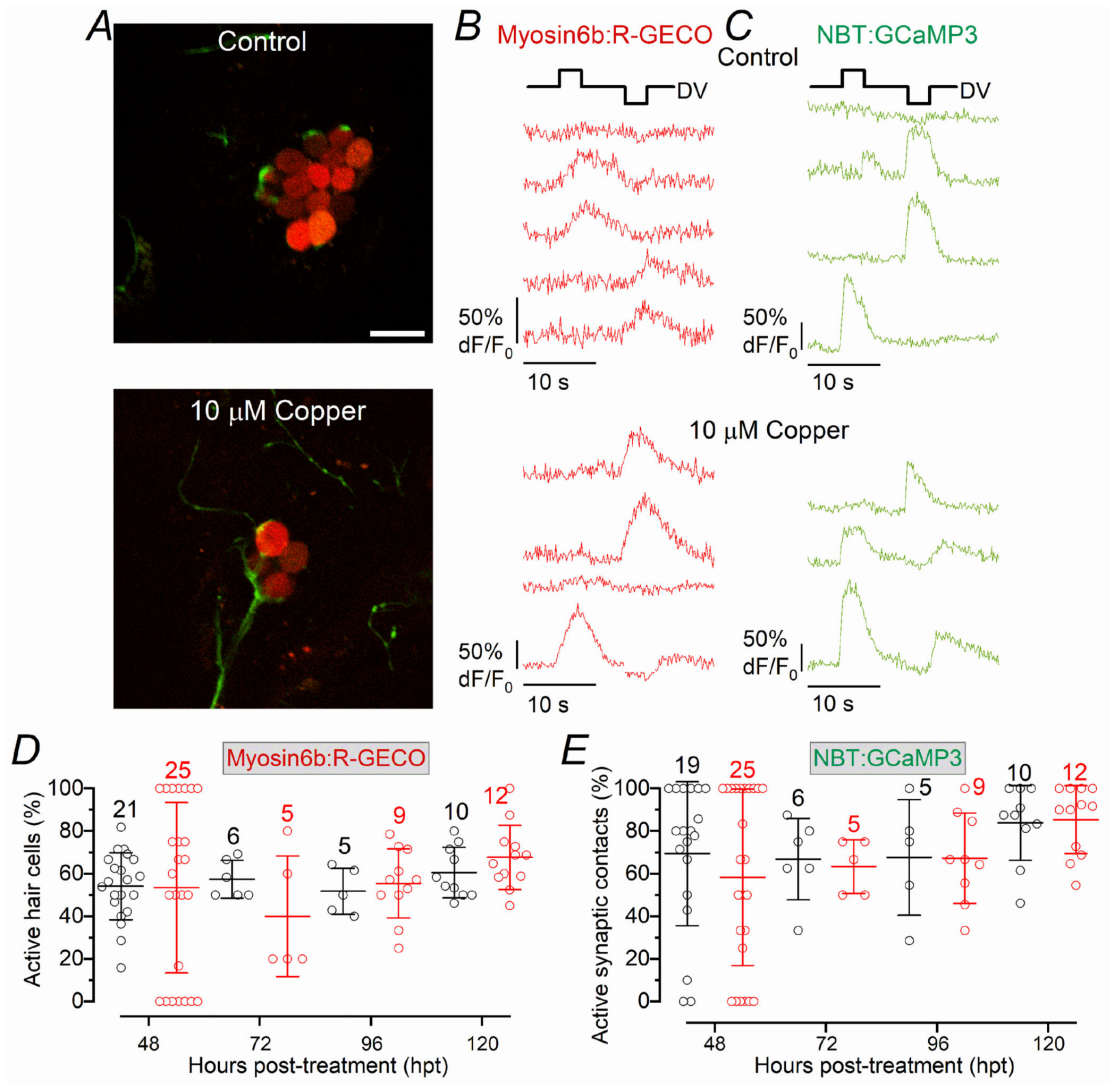
Synaptic activity in regenerating late-larval zebrafish hair cells ***A***, Images of a neuromast in a control (upper panel) and copper-treated (lower panel) 12 dpf zebrafish (*Tg(Myosin6b:R-GECO);Tg(NBT:GCaMP3)*), in which hair cells are label red and the afferent fibres/terminals in green. Scale bar: 10 μm. ***B*** and ***C***, Ca2+ responses in hair cells (R-GECO, ***B***) and afferent terminals (GCaMP3, ***C***). Saturating driver voltage displacing the cupula is shown above the recordings. Note that some hair cells respond to the either the excitatory or inhibitory cupula displacement depending on their polarity sensitivity. ***D***, Percentage of hair cells showing presynaptic Ca^2+^ responses during hair bundle stimulation in both (black) and copper-treated (red) 12 dpf zebrafish as a function of hpt (*P* = 0.7617, two-way ANOVA). The x-axis shows four time points (as hours post-treatments: hpt) following the application of copper at 12 dpf zebrafish. ***E***, Percentage of afferent synaptic terminals showing GCaMP3 responses during bundle stimulation as a function of hpt in control (black) and copper-treated (red) zebrafish (*P* = 0.6531, two-way ANOVA). In panels ***E*** and ***F***, the number of neuromasts tested is shown above the data; 2-3 neuromasts per zebrafish.

Despite the longer time course required to regenerate the lateral line in the late-larval zebrafish, the newly formed hair cells and their post-synaptic afferent terminals displayed Ca^2+^ responses when stimulated using the fluid-jet. Moreover, the proportion of active hair cells and afferent terminals was comparable to those in untreated zebrafish already at 48 hpt (Fig. 9A-E), as also found in early-larval zebrafish (Fig. 5E,F). This suggests that the newly regenerated hair cells can rapidly become functional even in older-larval zebrafish. Compared to early-larval zebrafish (Fig. 5F), the percentage of active post-synaptic afferent terminals within a neuromast in 14-17 dpf zebrafish increased to ~70% (Fig. 9E), which is comparable to previous measurement of synaptic activity in hair cells at 13 dpf (~50%: Zhang *et al*. 2018).

The above findings indicate that the complete functional and morphological regeneration of neuromasts in late-larval zebrafish requires 5 or more days post-treatment, which is substantially longer than that required for early-larval zebrafish.

### Afferent input is required for the regeneration of the neuromast

**Figure 10:**
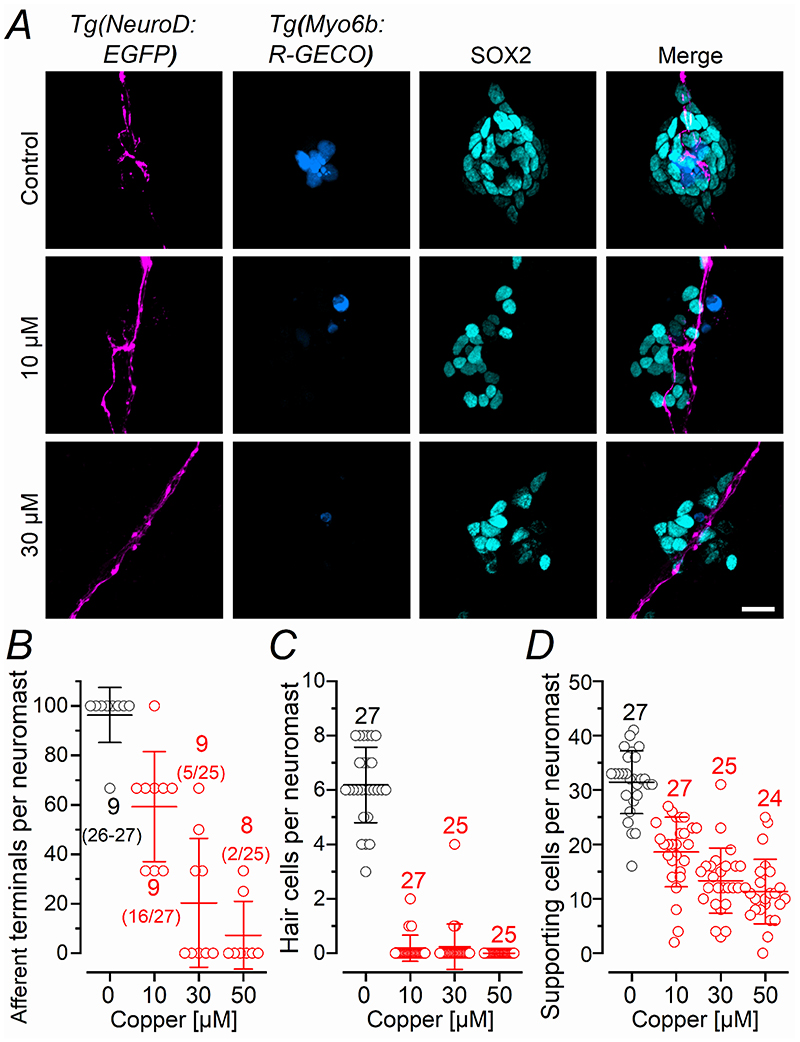
Afferent terminals and supporting cells are less sensitive to copper damage than hair cells ***A***, Confocal images showing the afferent fibres and terminals (magenta: *Tg(NeuroD:EGFP)* zebrafish line), hair cells (blue: *Tg(Myosin6b:R-GECO)* zebrafish line) and supporting cells (cyan: anti-Sox2 antibody) within a neuromast from control and copper-treated zebrafish (2 hours incubation with 10, 30 or 50 μM copper sulphate). Experiments were done using 3 dpf zebrafish. Scale bar: 10 μm. ***B-D***, Number of afferent terminals (***B***), hair cells (***C***) and supporting cells (***D***) per neuromast after the application of different concentrations of copper. The presence of afferent terminals was defined as an enlargement of the fibre within the neuromast region (presence of afferent terminals was scored as 100; absence with 0, which were then converted in % based on the number of neuromast investigated). For all three comparisons in panels ***B-D***, *P* < 0.0001, one-way ANOVA.

**Figure 11:**
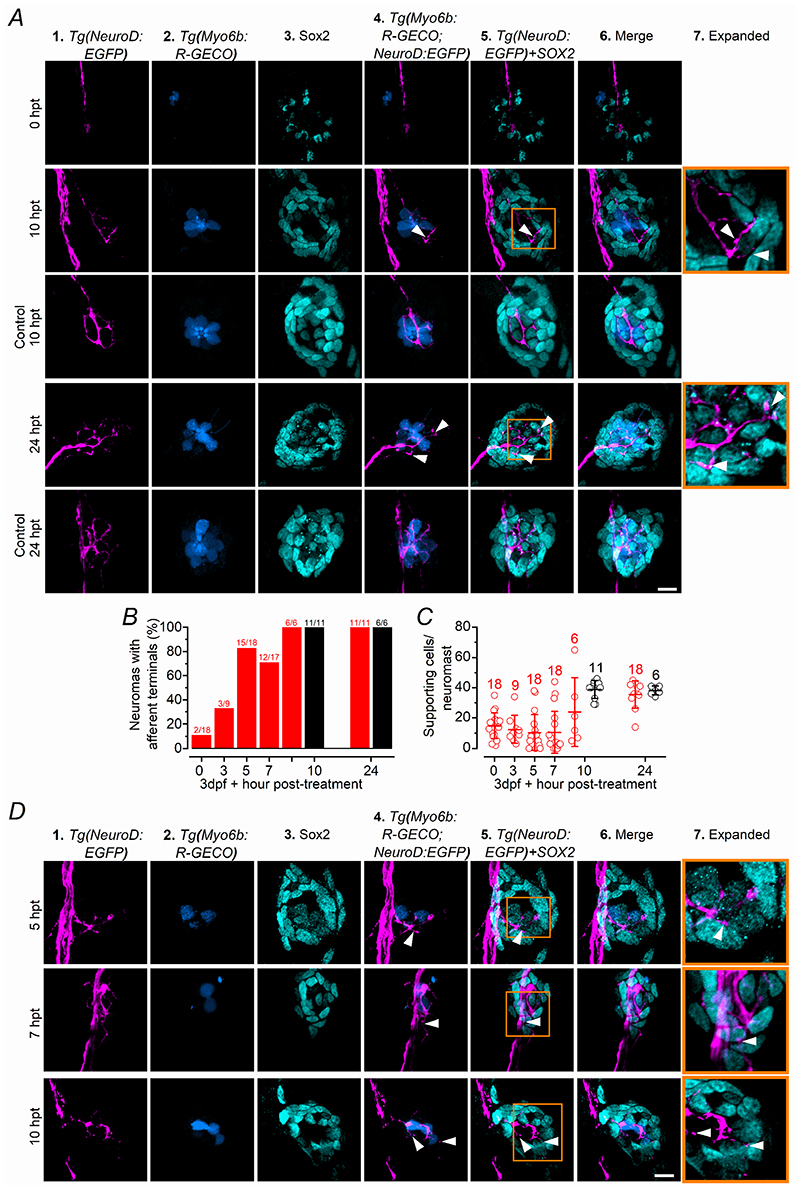
Afferent terminals within the regenerating neuromast colocalise with supporting cells ***A***, Confocal images showing the afferent fibres (magenta: **1**), hair cells (blue: **2**) and supporting cells (cyan: antibody anti-Sox2: **3**) within a neuromast from control and copper-treated zebrafish (2 hr incubation with 30 μM copper). The next three columns are merge images showing: afferent fibres and hair cells (**4**); afferent fibres and supporting cells (**5**); and afferent fibres, hair cells and supporting cells (**6**). The two images in the “expanded” column (**7**) highlight the presence of afferent terminals in close proximity to supporting cells. Experiments were done using 3 dpf *Tg(NeuroD:EGFP; Tg(Myosin6b:R-GECO)* zebrafish. Scale bar: 10 μm. ***B***, Percentage of neuromast showing afferent terminals as a function of hpt. Control neuromasts were only analyzed at 10 and 24 hpt. ***C***, Number of supporting cells per neuromast obtained in control (black) and copper-treated (red) zebrafish has a function of hpt. At 10 hpt, control vs copper: *P* = 0.1668; At 24 hpt, control vs copper: *P* = 0.9998, one-way ANOVA, Tukey’s post-test. ***D***, Confocal images as described in panel ***A***, for 5, 7 and 10 hpt neuromasts showing some additional examples of afferent terminals in the proximity of the supporting cells. Scale bar: 10 μm.

In the regenerating early- and late-larval neuromasts we found several newly formed afferent terminals in regions devoid of hair cells, as judged by the absence of the early hair cell marker myosin 6 (Figs. 6D, 8D, respectively). Therefore, we investigated whether the regenerating afferent terminals were initially targeting the supporting cells. Because the afferent fibres appear more resistant than hair cells to copper exposure, we first established the concentration required to fully abolish their protrusions. We found that 2 hr treatment with 30 or 50 μM copper removed the large majority of afferent terminals (Fig. 10A,B) and hair cells (Fig. 10A,C) with comparable effects (*P* = 0.5169 and *P* = 0.7612, respectively, one-way ANOVA, Tukey’s post-test). This range of copper concentrations also ablated around 50% of the supporting cells within each neuromast (Fig. 10A,D). Therefore, we treated *Tg(NeuroD:EGFP;Tg(Myosin6b:R-GECO)* early-larval zebrafish with 30 μM copper for 2 hrs, which were consequently fixed at different time points and immunostained with an antibody against Sox2, which is a transcription factor that labels the supporting cells within the neuromast (Hernández *et al*. 2007). Although the large majority of neuromasts were deprived of afferent terminals immediately after the copper treatment, by 10 hpt all neuromasts investigated displayed afferent terminals as found in control zebrafish (Fig. 11A,B). Indeed, the regeneration of the PLLg nerve and terminals within a neuromast has been shown to be independent from the presence of hair cells (Villegas *et al*. 2012). The number of Sox2-positive cells within each neuromast also became comparable to that of untreated zebrafish by 10 hpt (Fig. 11A,C). Additionally, several of the newly formed afferent terminals within the regenerating neuromasts were in close proximity to the supporting cells, especially within the first few hours after copper treatment, when only very few hair cells were present (Fig. 11D; see also Supplementary Movies 3,4).

**Figure 12:**
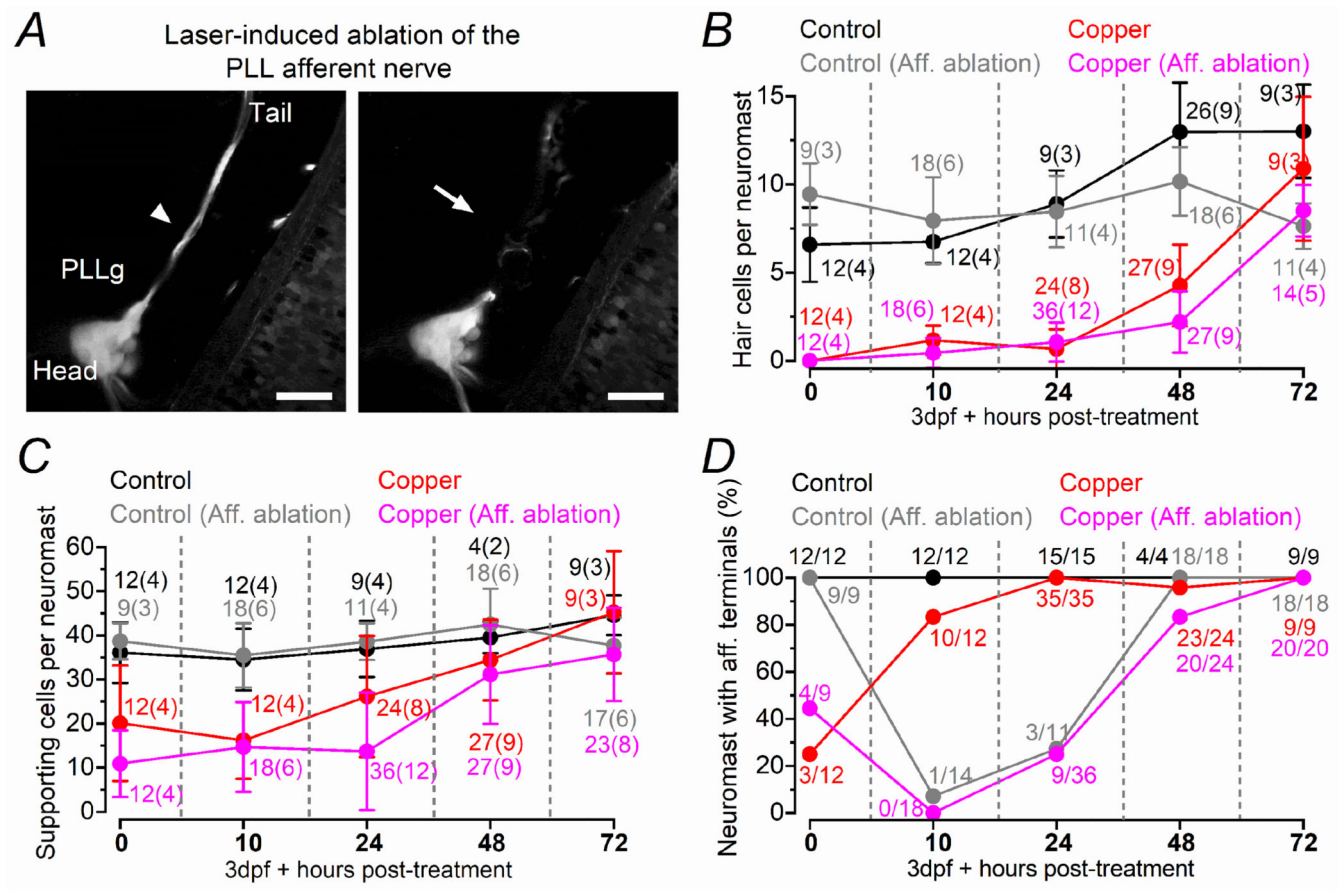
Afferent nerve regeneration is rapid and does not cause any substantial delay in the regeneration of the neuromast ***A***, Two-photon confocal images showing an image of the PLLg and its afferent fibres before (arrowhead: left) and after laser ablation (arrow, right). Scale bar: 30 μm. ***B***-***D***, ***B***, Number of hair cells per neuromast as a function of hpt. Zebrafish subjected to the ablation of the afferent nerves are indicated as: Control (Aff. Ablation) and Copper (Aff. Ablation). The experiment “Control (no Aff.)”, was performed to test whether the severance of the afferent nerves has any unforeseen effect on the untreated neuromasts. ***C***, Number of supporting cells per neuromast obtained under the same experimental conditions mentioned in panel ***B***. In panels ***B*** and ***C***, the number of neuromasts (zebrafish) tested is shown above the data points. ***D***, Percentage of neuromasts showing afferent protrusion/total neuromast investigated. Data in ***B-D*** were obtained by analysing confocal images obtained from zebrafish (3 dpf: *Tg(NeuroD:EGFP);Tg(Myosin6b:R-GECO*) that were treated for 2 hr with 30 μM copper sulphate (see Supplementary Figure 3).

**Figure 13:**
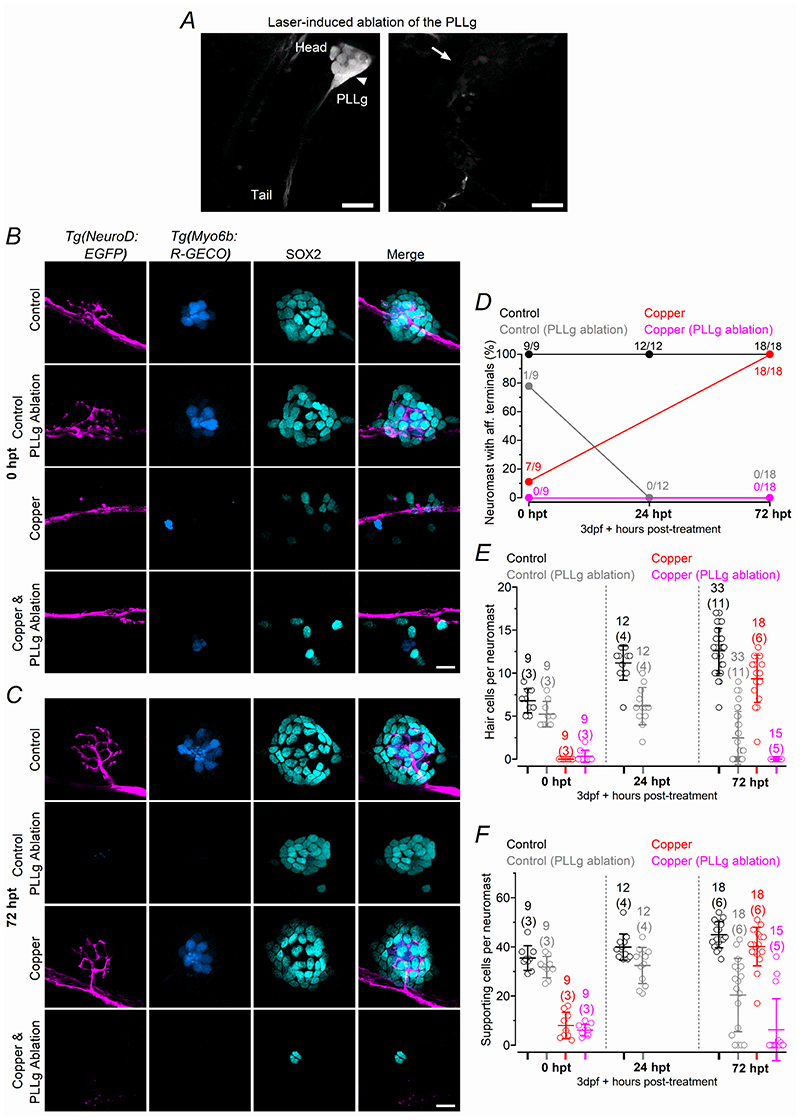
The PLLg is required for the regeneration of hair cells ***A***, Two-photon confocal images showing an image of the PLL before (arrowhead: left) and after laser ablation (of the PLLg arrows, right). Scale bar: 30 μm. ***B***, ***C***, Confocal images showing the afferent fibres (magenta), hair cells (blue) and supporting cells (cyan: antibody anti-Sox2) within a neuromast from control zebrafish (top panels, no-copper treated and no-PLLg ablated zebrafish), control-ablated zebrafish (second row of panels), copper-treated zebrafish (third row of panels) and copper treated zebrafish with ablated PLLg (bottom panels) at 0 hpt (***B***) and 72 hpt (***C***). Scale bar: 10 μm. Zebrafish (3 dpf: *Tg(NeuroD:EGFP); Tg(Myosin6b:R-GECO*)) were treated for 2 hr with 30 μM copper sulphate. The last column represents the merged images showing afferent fibres, supporting cells and hair cells. ***D***, Percentage of neuromasts showing afferent protrusion/total neuromast investigated. Zebrafish subjected to the ablation of the PLLg are indicated as: Control (PLLg Ablation) and Copper (PLLg Ablation). ***E***, Number of hair cells per neuromast as a function of hpt. The experiment “Control (PLLg Ablation)”, was performed to test whether the severance of the afferent nerves had any unforeseen effect on the untreated neuromasts. ***F***, Number of supporting cells per neuromast obtained under the same experimental conditions mentioned in panel ***E***. For additional statistical analysis see the “Statistical Summary” file. In panels ***E*** and ***F***, the number of neuromasts (zebrafish) tested is shown above the data points.

We next investigated whether the presence of the afferent terminals within the regenerating neuromast was required for the generation of new hair cells (Figs. 12, 13). Initially, we severed the afferent nerve downstream to the PLLg, but prior the first neuromast L1 (Fig. 12A, see Methods), after the application of 30 μM copper for 2 hrs. Immediately after copper treatment (with or without severed nerve: 0 hpt), the number of hair cells and supporting cells within each neuromast followed a comparable regeneration time course (Fig. 12B,C; see also Supplementary Fig. 3). However, since severed afferent nerves were able to regenerate relatively rapidly (Fig. 12D: see also Grant *et al*. 2005; Villegas *et al*. 2012), it prevented a reliable assessment of the possible role of the afferent input on the reappearance of both hair cells and supporting cells within the neuromast. We next ablated the entire PLLg (Fig. 13A), which completely prevented the regeneration of the afferent nerve leaving the neuromasts devoid of afferent input (Fig. 13B-D). In copper-treated zebrafish with the PLLg ablated, there was very little or no regeneration of hair cells (Fig. 13E, *P* < 0.0001 for copper vs copper-ablated, Sidak’s post-test, two-way ANOVA) and supporting cells (Fig. 13F, *P* < 0.0001) even at 72 hpt. Interestingly, control zebrafish with the ablated PLLg exhibited a progressive reduction in the number of hair cells (Fig. 13E, *P* < 0.0001, for control vs control-ablated, Sidak’s post-test, two-way ANOVA) and supporting cells (Fig. 13F, *P* < 0.0001) over the investigated time window. These findings indicate that the afferent input is required not only for the normal regeneration of the neuromast but also for its maintenance.

## Discussion

This study has shown that the firing rate of the spontaneous action potential activity in the PLLg of the zebrafish progressively increases over the first 6-8 dpf, with the afferent fibres displaying a higher firing rate from around 6 dpf (Fig. 1). The treatment of 3 dpf zebrafish with 10 μM copper ablates all hair cells from the neuromast, which then regenerate to a number comparable to untreated control zebrafish by 48-72 hpt (5-6 dpf, Fig. 2). A comparable time course of recovery was seen in the ability of the neuromasts to respond to activity-dependent stimuli, which were induced by the displacement of the mechanoelectrical transducer (MET) apparatus. Within each neuromast, the Ca^2+^ signals in both the hair cells and post-synaptic afferent terminals induced by the activation of the MET current, were comparable between copper-treated and control zebrafish by ~48 hpt (Fig. 5), a time when the induced APs in the PLLg neurons were also indistinguishable among the two experimental conditions (Fig. 4). Despite requiring 2-3 days to regenerate the full complement of hair cells within a neuromast, newly formed hair cells showed spontaneous glutamate release at their synapses and elicited action potential activity in the PLLg indistinguishable from those measured in untreated zebrafish by ~24 hpt (Fig. 3). This rapid acquisition of synaptic function was also consistent with the time course of regeneration of their synaptic machinery, defined by the colocalization of the CtBP puncta with the afferent projections (Fig. 6). At older ages (12-18 dpf), when the hair cells (Olt *et al*. 2014) and afferent fibres firing characteristics become more mature (Fig. 1), we found that the functional regeneration of the neuromast required much longer than in the >5 dpf zebrafish (Figs. 7-9). We also found that in both early- and late-larval zebrafish the afferent terminals in the regenerating neuromast appeared to make an initial contact with supporting cells (Figs. 8,11). Moreover, the removal of the afferent input to the neuromast substantially alter the normal regeneration of supporting cells and hair cells (Figs. 12, 13).

### Spontaneous firing activity in the lateral line system

Spontaneous electrical activity in the hair cell postsynaptic afferent fibres has previously been shown to occur not only in lower vertebrates (e.g. zebrafish: Weeg & Bass, 2002; Trapani & Nicolson, 2010; Liao & Haehmel, 2012; bullfrog: Keen & Hudspeth, 2006) but also in the mammalian system (e.g. guinea-pig: Manley & Robertson, 1976; mice: Sontag *et al*. 2009). This activity is known to be dependent upon the spontaneous release of glutamate-containing vesicles from the hair cells (Obholzer *et al*. 2008; Seal *et al*. 2008) via the activation of CaV1.3 Ca^2+^ currents (Platzer *et al*. 2008; Brandt *et al*. 2003; Trapani & Nicolson, 2010; Jeng *et al*. 2020). CaV1.3 Ca^2+^ channels have a relatively hyperpolarized activation (about -70 mV: Zampini *et al*. 2010; 2013), which is at or near to the resting membrane potential (*V*_m_) of hair cells in the zebrafish lateral line (Olt *et al*. 2014). Because the MET channels in the hair cells of the lateral line are partially open in the absence of external stimulation (Erickson *et al*. 2017), as also shown in other lower vertebrates and in mammals (e.g. Crawford *et al*. 1989; Holt *et al*. 1997; Corns *et al*. 2014), the standing inward depolarizing MET current directly contributes to the generation of the spontaneous action potential activity in the PLLg neurons (Trapani & Nicolson, 2011). Indeed, we found that the PLLg neurons, which carry the sensory input transduced by the hair cells in the neuromast, exhibit spontaneous action potential activity already at 2 dpf, which is when the resting MET current was first detected in these cells (Kindt *et al*. 2012).

The biophysical characteristics of this spontaneous firing activity in the afferent fibres of the lateral line has extensively been investigated below ~6 dpf (e.g. Trapani *et al*. 2011; Liao & Haehmel, 2012; Levi *et al*. 2015) and in the adult zebrafish (Weeg & Bass, 2002). One distinct property of this electrical activity is the large variability in its firing rate at all age-ranges investigated, which is likely to be due to several reasons, including: the size and age of the neurons (Liao & Haehmel, 2012), the number of neuromasts and hair cells of the same polarity that the neurons are contacting (Nagiel *et al*. 2008) and the possible differential modulation of the efferent system (Lunsford *et al*. 2019). Despite the relative similarity in the firing rate of the afferent neurons between larval and adult zebrafish, the presence of multiple patterns of APs appears to be only a characteristic of adult afferent neurons (Weeg & Bass, 2002). Here we demonstrated that a second neuronal population showing a higher firing rate and a more regular pattern is appearing from ~6 dpf, which became more prominent during the following days (Fig. 1), indicating that the lateral line is still maturing during the first week after fertilization. This is also consistent with previous data showing that the afferent fibres of the lateral line undergo extensive growth and reorganization (Haehnel *et al*. 2012) and that the biophysical properties of hair cells within a neuromast are still changing (Olt *et al*. 2014) during early-larval stages, all of which are essential for the fine-tuning and increased sensitivity to movement of adult zebrafish.

### Time course of functional regeneration in hair cells and afferent fibres

Hair cell regeneration in the zebrafish lateral line has primarily been investigated during the first few days post-hatching using morphological approaches. The application of ototoxic compounds, such as aminoglycosides or noise-induced damage (Holmgren *et al*. 2020), has been shown to cause the rapid loss of hair cells in the early-larval neuromasts, which is followed by their regeneration from surrounding supporting cells within ~48 hpt (e.g. Harris *et al*. 2003; Ma *et al*. 2008; Wibowo *et al*. 2011; Mackenzie & Raible, 2012). Our functional data from the early-larval neuromast further support these previous studies. The rapid recovery can also be visualised by the ability of larval zebrafish (<8 dpf) to regain normal escape and rheotaxis responses following aminoglycoside-induced hair cell death (within 48 hpt: McHenry *et al*. 2009; Suli *et al*. 2012). We also showed that despite the need for ~2 days to regain functional neuromasts following their damage, newly formed hair cells and their ribbon synapses are already functional by ~24 hpt, since they are able to drive spontaneous action potential activity in the PLLg neurons with a frequency and pattern comparable between treated and untreated zebrafish. Despite the similar number of hair cells present in the fully functional neuromasts after copper treatment at 6-8 dpf (~15 cells, Fig. 2E) and 12-18 dpf (~16 cells, Fig. 7B) stages, the latter older larval zebrafish require >120 hpt to functionally regenerate a neuromast, which is almost double that needed in early-larval zebrafish. The regeneration of hair cells from the lateral line of early-larval zebrafish has been shown to be mediated by supporting cells with distinct progenitor identities (Thomas & Raible, 2019). The possible change in the progenitor populations or mechanisms underpinning regeneration in older fish could explain their delayed regeneration capacity. However, a similar regenerative time window to that identified in late-larval zebrafish (>120 hpt) is required to fully regenerate the much larger number of hair cells present in the neuromast of a 3-year old fish (~148 hpt, Cruz *et al*. 2015). Therefore, the most likely and simpler explanation for the faster regeneration capability of the neuromasts in larval zebrafish is that during the first week post-fertilization, hair cells are still undergoing major developmental changes (Fig. 1; see also Olt *et al*. 2014), which occur in parallel to those responsible for cell regeneration.

Our morphological data show that the afferent fibres innervating the regenerating early- and late-larval neuromasts were seen to form enlarged terminals away from the newly formed hair cells, but in close proximity to supporting cells. It is possible that these supporting cells are targeted during the transdifferentiation into hair cells. Supporting cells divide symmetrically to give rise to two daughter hair cells (Meckenzie & Raible, 2012; López-Schier & Hudspeth, 2006). Since the regenerative capacity of the neuromast is an ongoing process, (e.g. Pinto-Teixeira *et al*. 2015) supporting cells have to be continuously replaced. Indeed, supporting cells from the neuromast of larval zebrafish can regenerate after copper-induced damage very rapidly and faster than hair cells (<24 hpt, Fig. 11). Although previous studies have shown that the initial development of the zebrafish lateral line neuromast does not require the presence of the PLLg nerve (Andermann *et al*. 2002; Grant *et al*. 2005; López-Schier & Hudspeth, 2005), our data indicate that the timely regeneration of the supporting cells and hair cells requires the presence of the afferent input. Since the afferent terminals seem to make initial contacts with supporting cells within a regenerating neuromast (Figs. 6, 8, 11), it is possible that they are promoting the proliferation or transdifferentiation of supporting cells into hair cells. Once the hair cells have regenerated, the presence of the afferent innervation has also been shown to be required for the correct localization of the ribbons at their pre-synaptic site (Suli *et al*. 2016). Interestingly, we also found that untreated zebrafish with ablated PLLg neurons undergo a progressive loss of hair cells and supporting cells, indicating that the afferent input is not only required for the timely regeneration of the neuromasts, but also for their maintenance, as shown in the adult zebrafish (Wada *et al*. 2013). A comparable role for the afferent input has also been identified in the maintenance and regeneration of the olfactory system of the *Xenopus laevis* larvae (Yoshino & Tochinai, 2006) and adult zebrafish (Byrd, 2000; Villanueva & Byrd-Jacobs, 2009; Paskin et al. 2011).

## Supplementary Material

 Video 3. Regenerating neuromast 1_morphology

Supplementary Figure

Video 1. Spontaneous release of glutamate from hair cells

Video 2. Induced calcium responses in hair cells and afferent terminals

Video 4. Regenerating neuromast 2_morphology

## Key Points Summary

- We investigated hair-cell regeneration in the zebrafish lateral line following the application of the ototoxic compound copper.
- In early-larval zebrafish (<10-days post-fertilization), regenerated hair cells drive action potentials (APs) in the afferent neurons 24-hours post-copper treatment (24 hpt).
- Full regeneration of the early-larval neuromasts, the organs containing the hair cells, requires ~48 hours due to the progressive addition of hair cells and synaptic refinement.
- In older larval zebrafish, regenerated hair cells are active and drive afferent APs by 48 hpt, which is comparable to larvae, but the functional recovery of their neuromasts require >120 hpt.
- Afferent terminals within the regenerating neuromast appear to initially contact supporting cells, and their complete ablation prevents the timely reappearance of supporting cells and hair cells.
- We conclude that the regeneration of zebrafish neuromasts is slower after the initial developmental stages, and that the afferent input plays a key role in driving this process.
