# Supplementary material for: Functional development and regeneration of hair cells in the zebrafish lateral line
Source: J Physiol. Author manuscript; Available in PMC 2021 Dec 22. (PMC7612129; doi:10.1113/JP281522)
Supplement: Supplementary Figure [file EMS140583-supplement-Supplementary_Figure.pdf]

Functional development and regeneration of hair cells in the zebrafish lateral line

Katherine Hardy, Ana E. Amariutei, Francesca De Faveri, Aenea Hendry, Walter Marcotti, Federico Ceriani

This documents contains Supplementary Figures 1

Supplementary Figure 1

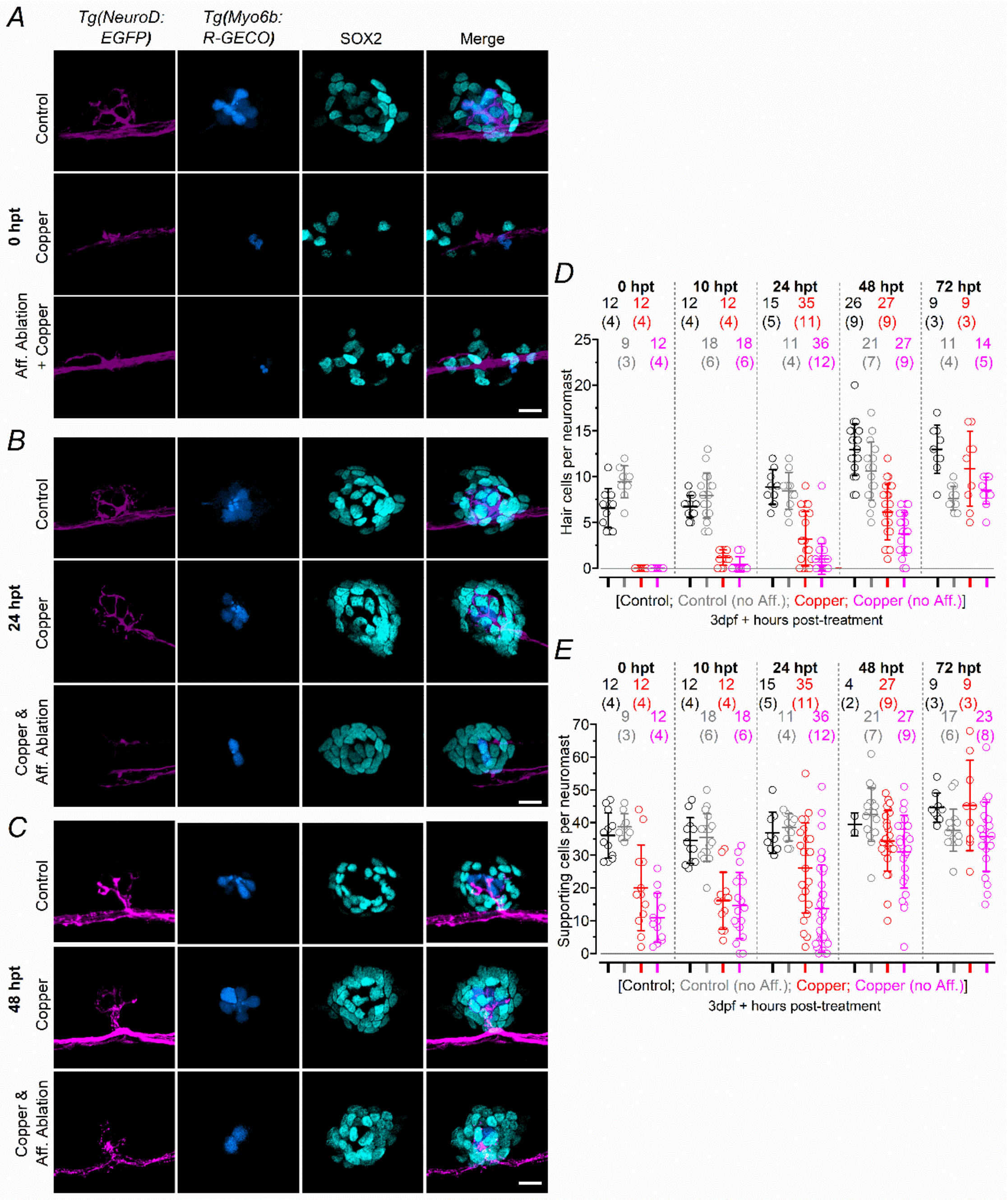

Supplementary Figure 1. Influence of nerve ablation on the regeneration of hair cells and supporting cells.

A-C, Confocal images showing the afferent fibres (magenta), hair cells (blue) and supporting cells (cyan: antibody anti-Sox2) within a neuromast from control (top panels), copper-treated (middle panels) and laser-ablated afferent neurons performed after copper treatment (bottom panels) at 0 hpt (***A***), 24 hpt (***B***) and 48 hpt (***C***). Scale bar: 10 µm. Zebrafish (3 dpf: *Tg(NeuroD:EGFP); Tg(Myosin6b:R-GECO)*) were treated for 2 hr with 30 µM copper sulphate. The last column represents the merged images showing afferent fibres, supporting cells and hair cells. ***D***, Number of hair cells per neuromast as a function of hpt. Zebrafish subjected to the ablation of the afferent nerves are indicated as: Control (no Aff.) and Copper (no Aff.). The experiment "Control (no Aff.)", was performed to test whether the severance of the afferent nerves has any unforeseen effect on the untreated neuromasts. ***E***, Number of supporting cells per neuromast obtained under the same experimental conditions mentioned in panel ***D***. In panels ***E*** and ***F***, the number of neuromasts (zebrafish) tested is shown above the data points.
